# Durability of neutralizing RSV antibodies following nirsevimab administration and elicitation of the natural immune response to RSV infection in infants

**DOI:** 10.1038/s41591-023-02316-5

**Published:** 2023-04-24

**Authors:** Deidre Wilkins, Yuan Yuan, Yue Chang, Anastasia A. Aksyuk, Beatriz Seoane Núñez, Ulrika Wählby-Hamrén, Tianhui Zhang, Michael E. Abram, Amanda Leach, Tonya Villafana, Mark T. Esser

**Affiliations:** 1 Translational Medicine, Vaccines & Immune Therapies, BioPharmaceuticals R&D, AstraZeneca, Gaithersburg, MD USA; 2 Biometrics, Vaccines & Immune Therapies, BioPharmaceuticals R&D, AstraZeneca, Madrid, Spain; 3 Clinical Pharmacology & Quantitative Pharmacology, R&D, AstraZeneca, Gothenburg, Sweden; 4 Data Sciences and Quantitative Biology, R&D, AstraZeneca, Gaithersburg, MD USA; 5 Clinical Development, Vaccines & Immune Therapies, Biopharmaceuticals R&D, AstraZeneca, Gaithersburg, MD USA; 6 Vaccines & Immune Therapies, Biopharmaceuticals R&D, AstraZeneca, Gaithersburg, MD USA

**Keywords:** Viral infection, Vaccines, Immunization

## Abstract

Nirsevimab is an extended half-life monoclonal antibody specific for the prefusion conformation of the respiratory syncytial virus (RSV) F protein, which has been studied in preterm and full-term infants in the phase 2b and phase 3 MELODY trials. We analyzed serum samples collected from 2,143 infants during these studies to characterize baseline levels of RSV-specific immunoglobulin G antibodies and neutralizing antibodies (NAbs), duration of RSV NAb levels following nirsevimab administration, the risk of RSV exposure during the first year of life and the infant’s adaptive immune response to RSV following nirsevimab administration. Baseline RSV antibody levels varied widely; consistent with reports that maternal antibodies are transferred late in the third trimester, preterm infants had lower baseline RSV antibody levels than full-term infants. Nirsevimab recipients had RSV NAb levels >140-fold higher than baseline at day 31 and remained >50-fold higher at day 151 and >7-fold higher at day 361. Similar seroresponse rates to the postfusion form of RSV F protein in nirsevimab recipients (68–69%) compared with placebo recipients (63–70%; not statistically significant) suggest that while nirsevimab protects from RSV disease, it still allows an active immune response. In summary, nirsevimab provided sustained, high levels of NAb throughout an infant’s first RSV season and prevented RSV disease while allowing the development of an immune response to RSV.

## Abstract (web summary)

The prolonged persistence at elevated levels of nirsevimab, an RSV-specific monoclonal antibody, likely accounts for the observed protection from severe disease throughout an RSV season, while it does not prevent the induction of a natural immune response in RSV-exposed infants.

## Main

RSV is the leading cause of acute lower respiratory tract infection (LRTI) in infants and young children^1,2^ and accounts for a substantial proportion of infant hospital admissions, healthcare resource utilization and high rates of infant mortality, particularly in developing countries^1,2^. RSV is a negative sense virus that codes for 11 proteins^3^ and circulates as two distinct serotypes (A and B)^4^ through an RSV season lasting approximately 5 months each winter in temperate climates. There are two major glycoproteins on the RSV virion envelope: the conserved fusion protein (F), which is present in prefusion (pre-F) and postfusion (post-F) forms, and the attachment protein (G), which exhibits genetic variability between RSV A and B subtypes^5–9^. RSV nucleocapsid (N) protein forms the helical ribonucleoprotein complex and protects the viral RNA from damage^10^.

Similar to other maternal antibodies, during the third trimester of pregnancy, RSV antibodies are transferred through the placenta and could provide some protection against RSV disease for approximately 3–6 months after birth^11–13^. However, levels of maternal antibodies against RSV at birth can be variable between infants and decline rapidly after birth^11–14^.

Nirsevimab is an anti-RSV monoclonal antibody, with an extended half-life in vivo (68.7 days (ref. ^15^)). It targets an antigenic region on the pre-F conformation of the F protein (which is conserved among circulating RSV A and B isolates) and thereby prevents RSV fusion with the host cell^8,16–20^. Most neutralizing activity from natural RSV infection is directed at the pre-F form of the F protein and thus forms a better target for monoclonal antibody development than the post-F form. The stabilization and structural characterization of the prefusion form of the F protein revealed that D25 (precursor to nirsevimab) binds to the highly neutralization-sensitive site Φ, which is exclusive to the pre-F surface^20^. Based on the high potency and extended half-life of nirsevimab, healthy neonates and infants were enrolled in two pivotal, global, double-blind, placebo-controlled studies to receive a single intramuscular (i.m.) dose of nirsevimab before the RSV transmission season for the prevention of RSV disease^15,21^. Nirsevimab reduced the incidence of medically attended RSV LRTI throughout an infant’s first RSV season, corresponding to an efficacy of 70.1% in healthy preterm infants in a phase 2b study (gestational age ≥29 to <35 weeks; median age at randomization 1.6 months)^21^ and 74.5% in healthy term and late preterm infants in the phase 3 MELODY study (gestational age ≥35 weeks; median age at randomization 2.6 months)^15^. Furthermore, a pooled analysis of infants who were administered nirsevimab at the approved dose regimen^22^ demonstrated an efficacy of 79.5% against medically attended RSV LRTI^23^. Here we present our analysis of results from the phase 2b and phase 3 MELODY studies with the following objectives: (1) characterize baseline maternal RSV antibody levels in preterm and full-term infants entering their first RSV season; (2) determine the level and duration of RSV NAb levels provided by nirsevimab; (3) investigate the incidence of clinical (symptomatic) and subclinical (asymptomatic) RSV infections in the first year of life; and (4) evaluate whether infants can mount a natural immune response against RSV in the presence of nirsevimab. For the phase 2b study (NCT02878330), this was a post hoc analysis and data were analyzed after completion of the study. For the MELODY study (NCT03979313), this was a prespecified exploratory analysis with a data cut-off of 9 August 2021.

## Results

### Disposition, demographics and baseline RSV antibody levels

**Extended Data Fig. 1:**
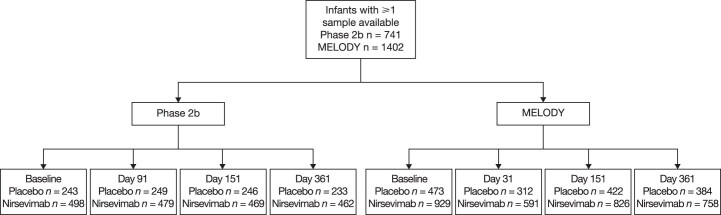
Disposition of infants who provided serum samples during the study. *n* denotes population with ≥1 sample available for testing at any time point for NAb. *n*, number of infants; NAb, neutralizing antibody.

Of 1,453 infants randomized in the phase 2b study and 1,490 randomized in the MELODY primary cohort, 741 and 1,402 infants, respectively, had baseline serum samples available. Of these, baseline antibody measurements were obtained from 498 infants randomized to nirsevimab and 243 infants randomized to placebo in the phase 2b study, along with measurements from 929 infants randomized to nirsevimab and 473 infants randomized to placebo in MELODY (Extended Data Fig. 1). Baseline demographics were similar in both treatment groups and between studies, with the exception of different gestational age (Supplementary Table 1). Mean age at randomization was 3.4 months in the phase 2b study and 3.0 months in MELODY for the population in this analysis.

**Fig. 1:**
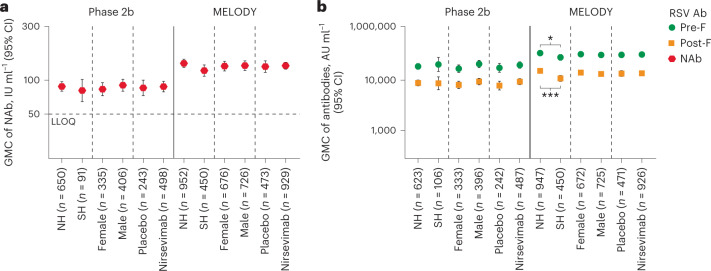
Baseline RSV NAb and antibody levels by hemisphere, sex and treatment. **a**, GMC of RSV NAb. **b**, GMCs of IgG antibodies pre-F and post-F (AU ml^−1^). *P < 0.05, ***P < 0.001. Data are presented as GMCs ± 95% CIs, which were calculated assuming log normal distribution. Two-sided *P* values were calculated based on the *F* statistic from analysis of variance (ANOVA), without adjustment. GMCs of NAb, pre-F and post-F were significantly higher in MELODY than in the phase 2b study (all *P* < 0.001); however, only the differences in GMC between hemispheres in MELODY were statistically significant (NAb, *P* = 0.0486; pre-F, *P* = 0.0292; post-F, *P* < 0.0001). *n*, number of infants; NH, Northern Hemisphere; SH, Southern Hemisphere.

**Fig. 2:**
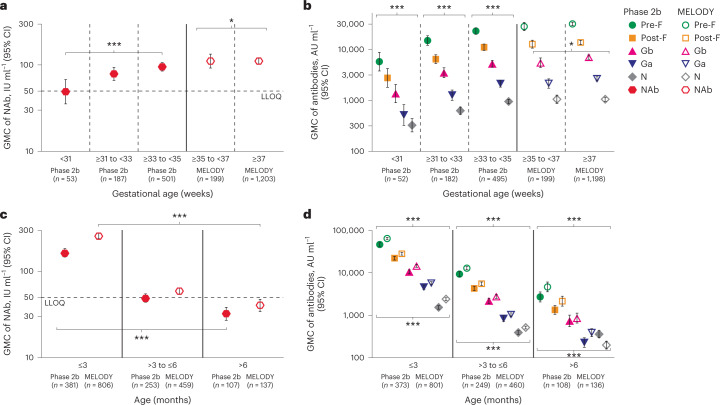
Baseline RSV-specific NAb levels. **a**, GMC of NAb by gestational age. **b**, GMC of RSV pre-F, post-F, Ga, Gb and N IgG antibodies by gestational age. **c**, GMC of NAb by infant age. **d**, GMC of pre-F, post-F, Ga, Gb and N IgG antibodies by infant age. *P < 0.05, ***P < 0.001. Data are presented as GMCs ± 95% CIs, which were calculated assuming log normal distribution. Two-sided *P* values were calculated based on the *F* statistic from ANOVA, without adjustment. In **a**, *P* = 0.0005 and *P* = 0.0274 for the phase 2b study and MELODY, respectively. In **b**, differences between groups within the phase 2b study were statistically significant (all *P* < 0.0001); for MELODY, only Gb was statistically different (*P* = 0.0406). In **c**, *P* < 0.0001 for both the phase 2b study and MELODY. In **d**, differences between groups within the phase 2b study and MELODY were statistically significant (all *P* < 0.0001).

**Extended Data Fig. 2:**
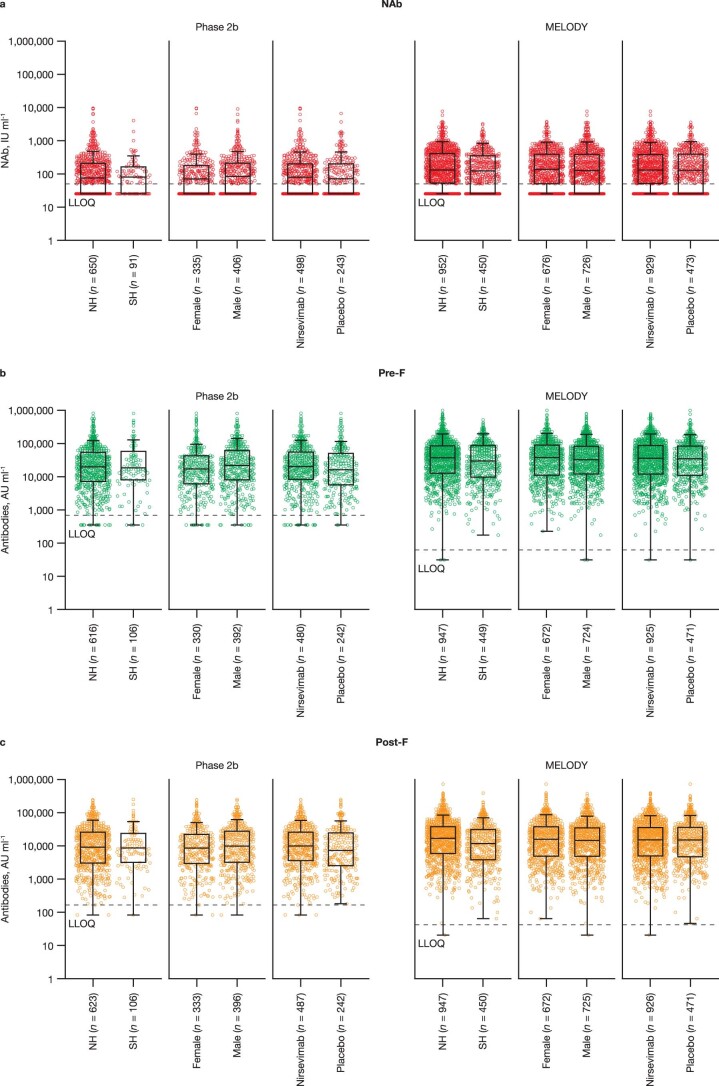
Baseline RSV NAb and antibody levels by hemisphere, sex, and treatment (AU ml^-1^). **a**, GMC of RSV NAb. **b**, GMC of pre-F. **c**, GMC of post-F. The box is bounded by the 25th and 75th percentiles; the line within the box represents the median. The whiskers represent 1.5 x IQR. No statistically significant differences between groups within the phase 2b study and MELODY were observed. AU ml^-1^, arbitrary units per milliliter; CI, confidence interval; F, fusion protein; GMC, geometric mean concentration; IgG, immunoglobulin G; IQR, interquartile range; IU ml^-1^, International Units per milliliter; LLOQ, lower limit of quantification; *n*, number of infants; NAb, neutralizing antibody; NH, Northern Hemisphere; RSV, respiratory syncytial virus; SH, Southern Hemisphere.

**Extended Data Fig. 3:**
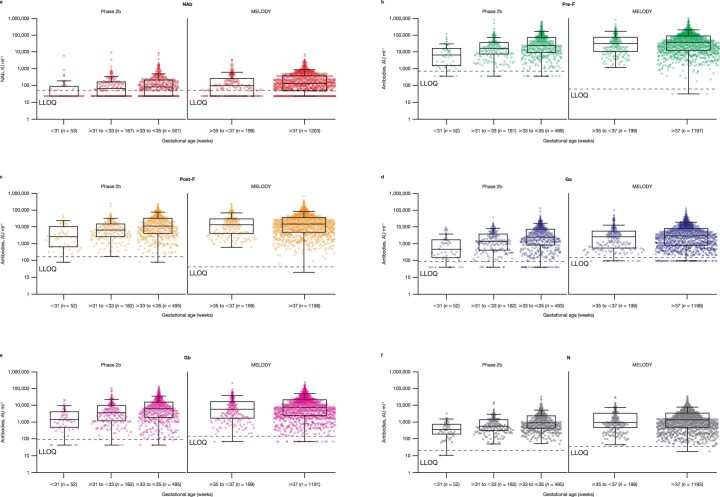
Baseline RSV-specific NAb and antibody levels by gestational age. **a**, GMC of NAb. **b**, GMC of pre-F. **c**, GMC of post-F. **d**, GMC of Ga. **e**, GMC of Gb. **f**, GMC of N. The box is bounded by the 25th and 75th percentiles; the line within the box represents the median. The whiskers represent 1.5 x IQR. AU ml^-1^, arbitrary units per milliliter; CI, confidence interval; F, fusion protein; Ga, attachment protein G RSV subtype A; Gb, attachment protein G subtype B; GMC, geometric mean concentration; IgG, immunoglobulin G; IQR, interquartile range; IU ml^-1^, International Units per milliliter; LLOQ, lower limit of quantification; *n*, number of infants; N, nucleocapsid N; NAb, neutralizing antibodies; RSV, respiratory syncytial virus.

**Extended Data Fig. 4:**
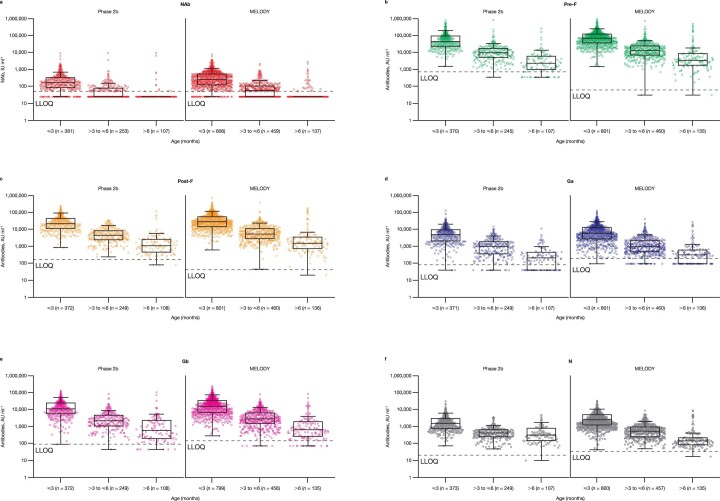
Baseline RSV-specific NAb and antibody levels by infant age. **a**, GMC of NAb. **b**, GMC of pre-F. **c**, GMC of post-F. **d**, GMC of Ga. **e**, GMC of Gb. **f**, GMC of N. The box is bounded by the 25th and 75th percentiles; the line within the box represents the median. The whiskers represent 1.5 x IQR. AU ml^-1^, arbitrary units per milliliter; CI, confidence interval; F, fusion protein; Ga, attachment protein G RSV subtype A; Gb, attachment protein G subtype B; GMC, geometric mean concentration; IgG, immunoglobulin G; IU ml^-1^, International Units per milliliter; IQR, interquartile range; LLOQ, lower limit of quantification; *n*, number of infants; N, nucleocapsid N; NAb, neutralizing antibodies; RSV, respiratory syncytial virus.

Since infants in both studies were enrolled before the start of their first RSV season, baseline antibody levels were considered to be maternal RSV antibodies and not due to a previous RSV exposure. As expected, baseline RSV NAb and RSV pre-F and post-F immunoglobulin G (IgG) antibody levels were similar in both studies across all subgroups (adjusted to infant postnatal age at baseline) (Fig. 1 and Extended Data Fig. 2). RSV NAb levels and RSV pre-F, post-F, attachment protein subtype A (Ga), attachment protein subtype B (Gb) and N IgG antibody levels were lower in the preterm infants enrolled in the phase 2b study than in late preterm and full-term infants enrolled in the MELODY study (Fig. 2 and Supplementary Table 2), although it should be noted that infants ranged in birth age from 1 day to 11 months at study entry in both studies, irrespective of gestational age. Of note, lower baseline antibody levels were observed in infants with gestational age <31 weeks compared with infants with gestational age >35 weeks, regardless of age at study entry (Fig. 2a,b and Extended Data Figs. 3 and 4). In addition, levels of RSV NAb, along with RSV pre-F, post-F, Ga, Gb and N IgG antibody levels decreased as infant age increased, with the lowest baseline RSV antibody levels found in infants >6 months of age in both studies (Fig. 2c,d and Extended Data Figs. 3 and 4).

**Fig. 3:**
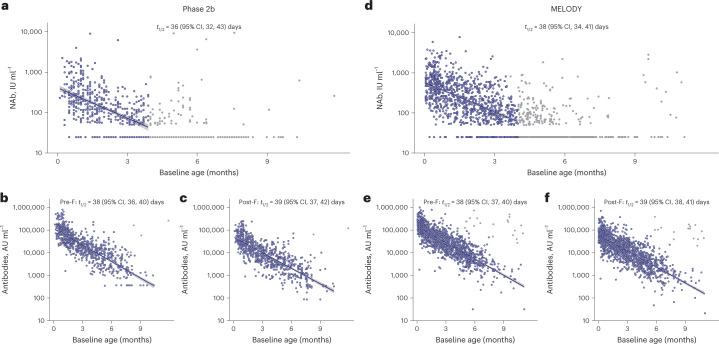
Half-life of RSV NAbs based on infant age at randomization. **a**, Phase 2b study NAbs. **b**, Phase 2b study pre-F IgG antibodies. **c**, Phase 2b study post-F IgG antibodies. **d**, MELODY NAbs. **e**, MELODY pre-F IgG antibodies. **f**, MELODY post-F IgG antibodies. Blue circles denote data included in the analysis; gray circles denote data that were excluded (as described in Methods section). The gray band surrounding each line represents the 95% CI*.* *t*_½_, half-life.

RSV maternal antibody half-life was estimated for each study, based on pooled infant data of RSV antibody levels at baseline (before dosing and before the start of the RSV season) and age at randomization. RSV NAb half-life was calculated to be 36 days (95% confidence interval (95% CI) 32, 43 days) for preterm infants in the phase 2b study and 38 days (95% CI, 34, 41 days) for infants in the MELODY study (Fig. 3a,d); RSV pre-F (38 days; phase 2b, 95% CI, 36, 40 days; MELODY, 95% CI, 37, 40 days) and post-F (39 days; phase 2b, 95% CI, 37, 42 days; MELODY, 95% CI, 38, 41 days) IgG antibody half-lives were similar in both studies (Fig. 3b,c,e,f). Of note, baseline RSV pre-F and post-F antibody levels varied more than 1,000-fold (phase 2b geometric mean concentration (GMC) range: pre-F 348.0–798, 416.0, post-F 82.5–246, 314.0; MELODY: pre-F 31.0–974,132.0, post-F 20.5–716,043.0) and few infants had pre-F, post-F, Ga, Gb and N antibody levels below the lower limits of quantification (pre-F: 2.6% and 0.1%; post-F: 0.4% and 0.1%; Ga: 5.6% and 4.8%; Gb: 1.1% and 1.3%; N: 0.1% and 0.1% for phase 2b and MELODY, respectively). In contrast, 38% of preterm infants and 25% of late-term and full-term infants had RSV NAb levels below the lower limit of quantitation at baseline across phase 2b and MELODY, respectively.

### Comparison of antibody profiles with and without RSV

Following administration of nirsevimab, fold rise from baseline in RSV NAb levels was calculated for each post-baseline visit and each participant; confidence intervals for geometric mean fold rise (GMFR) were calculated assuming a log normal distribution.

Overall (regardless of an RSV infection), we observed GMFR of 149 (95% CI, 131, 170) in RSV NAb levels from baseline, with a GMC of 134 international units per milliliter (IU ml−1) (95% CI, 125, 143) to the first sample collection timepoint at day 31 in the MELODY study (GMC 19,737 IU ml^−1^; 95% CI, 18,684, 20,849) and a GMFR of 94 (95% CI, 81, 109) from baseline (GMC 87 IU ml^−1^; 95% CI, 79, 95) to the first sample collection timepoint at day 91 in the phase 2b study (GMC 8,479 IU ml^−1^; 95% CI, 7,712, 9,322). At day 151 (typical length of an RSV season and timing for endpoint collection in both studies), nirsevimab recipients still exhibited RSV NAb levels higher than baseline, with GMFR of 53 (95% CI, 46, 62) in phase 2b, and 51 (95% CI, 46, 56) in the MELODY study. RSV NAb levels remained at least sevenfold higher than baseline through day 361 (phase 2b GMFR 8; 95% CI, 7, 10; MELODY GMFR 7; 95% CI, 6, 8).

**Fig. 4:**
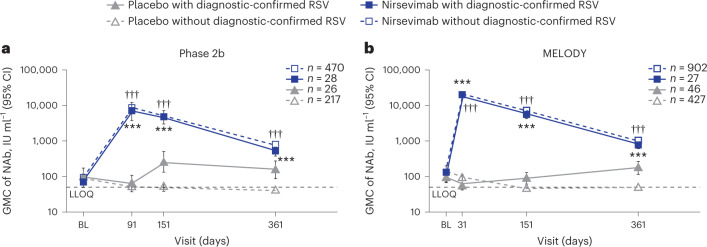
RSV NAb GMC through day 361 by treatment and medically attended, diagnostic-confirmed RSV infection. **a**, Phase 2b study NAbs. **b**, MELODY study NAbs. ***P < 0.001, nirsevimab versus placebo with diagnostic-confirmed RSV; ^†††^*P* < 0.001, nirsevimab versus placebo without diagnostic-confirmed RSV. *n* denotes number of infants who had a serum sample available at baseline. Data are presented as GMCs ± 95% CIs, which were calculated assuming log normal distribution. Two-sided *P* values were calculated based on the *F* statistic from ANOVA, without adjustment. In **a**, all were *P* < 0.0001, except for day 361 with diagnostic-confirmed RSV, which was *P* = 0.0005. In **b**, all were *P* < 0.0001. BL, baseline.

By day 361, RSV NAb levels in placebo recipients without a diagnostic-confirmed RSV infection (central or local test) decreased over time with a GMFR < 1, whereas in placebo recipients with a diagnostic-confirmed RSV infection there was a GMFR of 1 (95% CI, 0.5, 2) in the phase 2b study and a GMFR of 2 (95% CI, 1, 4) in MELODY (Extended Data Table 1). At day 361, most placebo recipients without a diagnostic-confirmed RSV infection during the studies had low to unmeasurable RSV NAb levels in the phase 2b study and in MELODY, while nirsevimab recipients without a diagnostic-confirmed RSV infection had RSV NAb GMCs of 757 IU ml^−1^ (95% CI, 702, 816) and 979 IU ml^−1^ (95% CI, 914, 1,048), respectively (Fig. 4). This corresponded to more than 19-fold higher geometric mean RSV NAb levels at day 361 in nirsevimab recipients versus placebo recipients without a diagnostic-confirmed RSV infection in both studies (Extended Data Table 1).

**Fig. 5:**
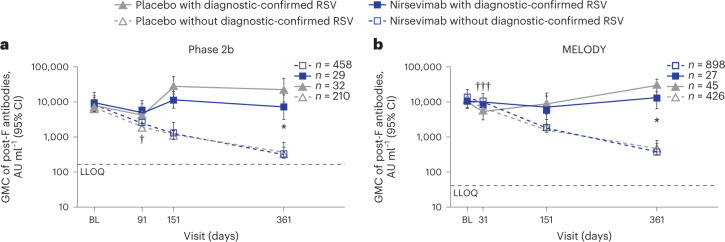
RSV post-F antibody GMC through day 361 by treatment and medically attended, diagnostic-confirmed RSV infection. **a**, Phase 2b study post-F IgG antibodies. **b**, MELODY study post-F IgG antibodies. *P < 0.05, nirsevimab versus placebo with diagnostic-confirmed RSV; ^†^*P* < 0.05, ^†††^*P* < 0.001, nirsevimab versus placebo without diagnostic-confirmed RSV. *n* denotes number of infants who had a sample available at baseline. Data are presented as GMCs ± 95% CIs, which were calculated assuming log normal distribution. Two-sided *P* values were calculated from the *F* statistic from ANOVA, without adjustment. **a**, At day 91 without diagnostic-confirmed RSV, *P* = 0.0227, and at day 361 with diagnostic-confirmed RSV, *P* = 0.0458. **b**, At day 31 without diagnostic-confirmed RSV, *P* < 0.0001, and at day 361 with diagnostic-confirmed RSV, *P* = 0.0391.

**Extended Data Fig. 5:**
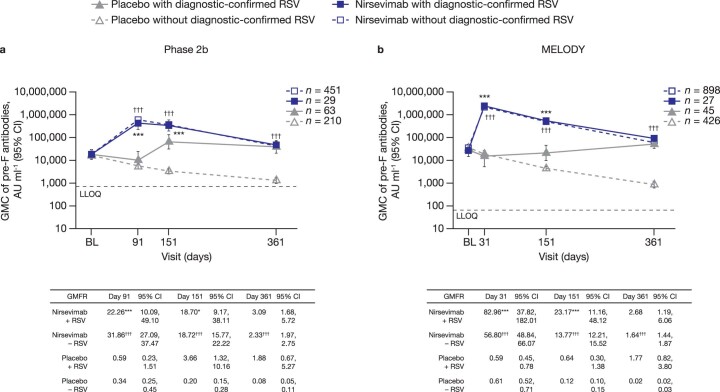
RSV pre-F IgG antibody GMC and GMFR through day 361 by treatment and medically attended diagnostic-confirmed infection. **a**, Phase 2b study pre-F antibodies. **b**, MELODY study pre-F antibodies. *n* denotes the number of infants at day 361. *P < 0.05, ***P < 0.001, nirsevimab vs placebo with diagnostic confirmed RSV; ^†††^*P* < 0.001 nirsevimab vs placebo without diagnostic confirmed RSV. Two-sided p-values were calculated based on the F statistic from ANOVA, without adjustment. AU ml^-1^, arbitrary units per milliliter; BL, baseline; CI, confidence interval; F, fusion protein; GMC, geometric mean concentration; GMFR, geometric mean fold rise; IgG, immunoglobulin G; LLOQ, lower limit of quantification; *n*, number of infants; RSV, respiratory syncytial virus.

**Extended Data Fig. 6:**
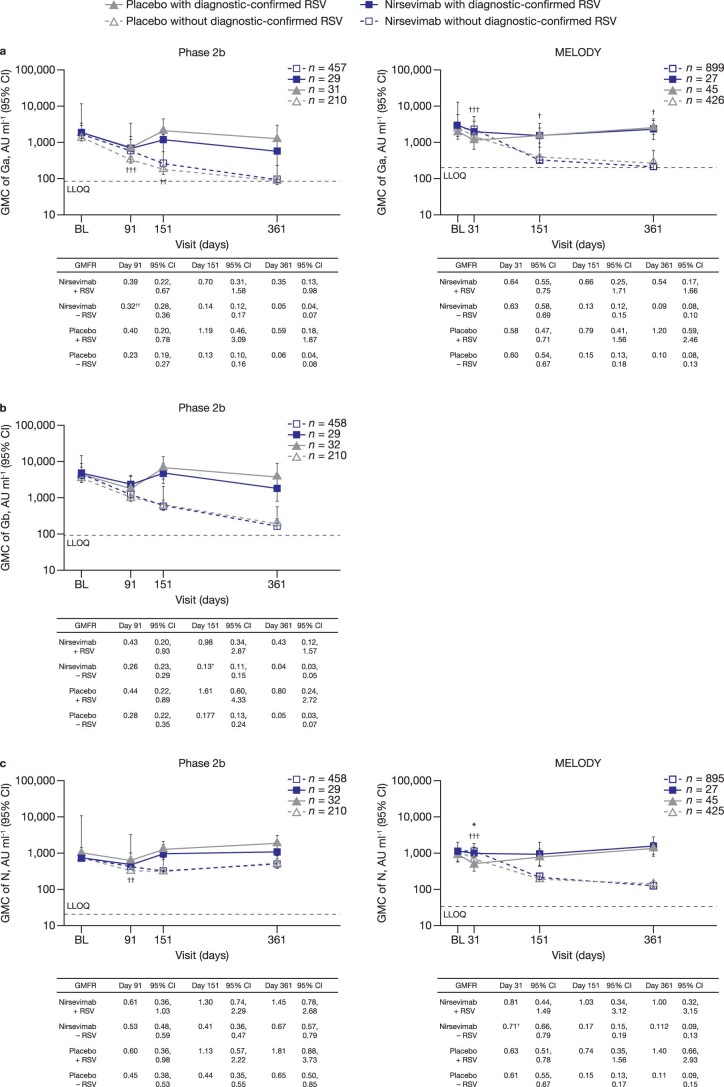
RSV GMC of IgG antibody levels through day 361 by treatment and medically attended diagnostic-confirmed infection. **a**, Ga. **b**, Gb. **c**, N. *P < 0.05, nirsevimab vs placebo with diagnostic-confirmed RSV; ^†^*P* < 0.05, ^††^*P* < 0.01, ^†††^*P* < 0.001 nirsevimab vs placebo without diagnostic-confirmed RSV. Data were unavailable for Gb for MELODY. The CIs for GMC were calculated assuming log normal distribution. Two-sided *P* values were calculated based on the F statistic from ANOVA, without adjustment. AU ml^-1^, arbitrary units per milliliter; CI, confidence interval; F, fusion protein; Ga, attachment protein G RSV subtype A; Gb, attachment protein G subtype B; GMC, geometric mean concentration; IgG, immunoglobulin G; LLOQ, lower limit of quantification; *n*, number of infants; N, nucleocapsid N; NAb, neutralizing antibodies; RSV, respiratory syncytial virus.

Further analyses were performed to quantitate specific RSV pre-F and post-F antibody levels post-baseline using data from the RSV multiplex serology assay. As nirsevimab is a NAb that binds to pre-F protein, it cannot be distinguished from pre-F IgG antibodies generated following an adaptive immune response; therefore, the GMC levels and GMFR follow the same pattern as observed for RSV NAb levels after administration of nirsevimab (Extended Data Fig. 5). In contrast, as RSV post-F antibody can only come from maternal transfer or an infant’s adaptive immune response following an RSV exposure, levels were broadly similar between nirsevimab and placebo recipients over time (Fig. 5), declining in infants without diagnostic-confirmed RSV infections but increasing in infants with a diagnostic-confirmed RSV infection in both nirsevimab and placebo recipients. This demonstrates that post-F antibody levels could be used to determine RSV exposure in infants who received nirsevimab. A similar pattern was observed with Ga, Gb and N levels (Extended Data Fig. 6). However, there were some variations between the two studies in participants with a diagnostic-confirmed RSV infection (Fig. 5). In phase 2b placebo recipients, there was an increase in RSV post-F GMC antibody levels between day 91 and day 151 compared with baseline, before gradually decreasing (Fig. 5a and Extended Data Table 2), while in MELODY post-F GMC antibody levels increased between day 151 and day 361, which corresponded with the delayed RSV season in the Southern Hemisphere observed in 2020 as a result of the COVID-19 pandemic (Fig. 5b and Extended Data Table 2). Of note, at day 361, post-F GMC antibody levels in participants with diagnostic-confirmed RSV were statistically higher in placebo (phase 2b *n* = 32, MELODY *n* = 45) versus nirsevimab recipients (phase 2b *n* = 29, MELODY *n* = 27) in both studies (both *P* < 0.05; Fig. 5).

A similar pattern was observed with Ga, Gb and N levels (Extended Data Fig. 6).

### Seroresponse by diagnostic-confirmed RSV infection

**Fig. 6:**
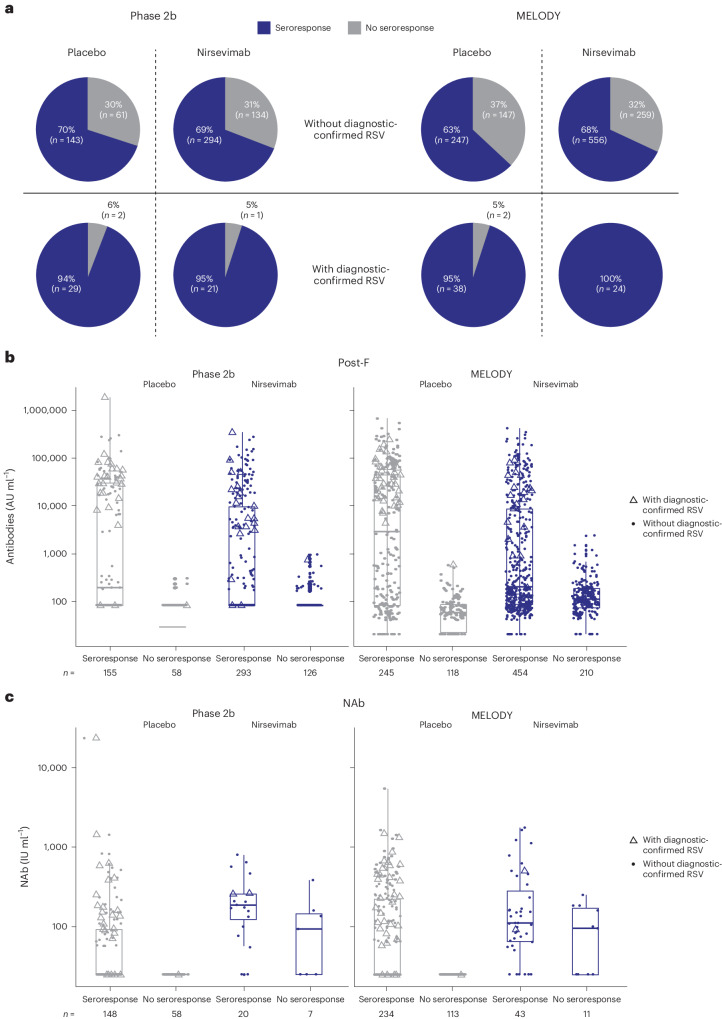
RSV seroresponse by treatment and medically attended, diagnostic-confirmed RSV LRTI. **a**, Proportion of participants with a seroresponse. **b**, RSV post-F antibody levels at day 361. **c**, RSV NAb level at day 361. The graphs show the subpopulation of participants with available data, for example, those who had a baseline sample and a day 151 and/or day 361 sample. Infants were defined as having a seroresponse if the RSV post-F antibody fold-change from baseline was above the respective cut point (>0.07 at day 151 or >0.02 at day 361; Supplementary Information Section 1). The box is bounded by the 25th and 75th percentiles; the line within the box represents the median. The whiskers represent 1.5 × interquartile range. MA, medically attended; *n*, number of infants who had a baseline sample and a day 151 and/or day 361 sample.

**Extended Data Fig. 7:**
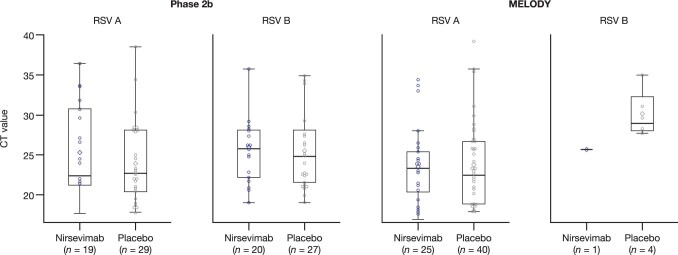
Viral load by RSV subtype based on central RT-PCR testing (ITT population). **a**, Phase 2b study. **b**, MELODY study. The box is bounded by the 25th and 75th percentiles; the line within the box represents the median. The whiskers represent 1.5 x IQR. CT, cycle threshold; IQR, interquartile range; ITT, intent-to-treat; *n*, number of infants; RSV, respiratory syncytial virus; RT-PCR reverse transcriptase polymerase chain reaction.

Of those infants with a confirmed RSV infection, there was no difference in viral load or RSV subtype between nirsevimab and placebo (Extended Data Fig. 7). RSV post-F antibody measurements in samples at baseline, day 151 and day 361 from infants who had a diagnostic-confirmed RSV infection were used to determine a statistical cut-point method to define seroresponse. The criteria for an RSV seroresponse were determined to be >0.07-fold-change at day 151 or >0.02 at day 361 in RSV post-F antibody levels from baseline (Methods). Based on these criteria, Fig. 6a shows that seroresponse rates of participants who had a medically attended, diagnostic-confirmed RSV infection were similar across studies, regardless of whether they received nirsevimab or placebo (94–100%); infants without a medically attended RSV LRTI had seroresponse rates of 63–70%. More specifically, among infants who did not have a medically attended, diagnostic-confirmed RSV infection, 70% and 63% of placebo recipients in the phase 2b and MELODY studies and 69% and 68% of the nirsevimab recipients had a seroresponse, respectively. These data suggest that the nirsevimab and placebo recipients were exposed equally to RSV. Of note, an analysis of the phase 2b and MELODY trials found that events of RSV infection occurred across both studies^23^.

### NAb response following RSV exposure

A key question raised by these results is whether nirsevimab affected the NAb response to RSV. To address this question, we examined the day 361 RSV NAb levels in nirsevimab recipients with undetectable nirsevimab levels who had either a diagnostic-confirmed RSV infection or an RSV exposure based on a post-F antibody seroresponse (Fig. 6b). Interestingly, the RSV NAb levels were similar or slightly higher in nirsevimab recipients, suggesting that nirsevimab recipients mounted an NAb response following infection (Fig. 6c). These data suggest that, akin to maternal antibodies, nirsevimab provides protection against disease, but still allows the infant immune system to elicit NAbs to RSV.

## Discussion

An ideal preventative solution for newborns would protect them from RSV disease during the initial period after birth when their immature immune systems are unable to generate an active response. The prophylaxis should protect across an entire RSV season, but not hinder infants’ adaptive immune systems (once able) from responding to subsequent exposures to RSV; we undertook this study to assess these characteristics of nirsevimab. Analysis from two randomized, placebo-controlled studies, which assessed the efficacy of nirsevimab in preventing medically attended RSV LRTI, found that baseline serum RSV NAb levels were higher in the MELODY study, likely due to the older gestational age providing additional time for transfer of maternal antibodies in this cohort (Fig. 1). Similarly, the correlation between gestational age and RSV antibody levels at baseline (Fig. 2) was consistent with previous reports that maternal antibodies are transferred later in the third trimester and may explain why premature infants are at increased risk of RSV disease^12,24–26^. Baseline RSV pre-F and post-F antibody levels differed by as much as 1,000-fold (Fig. 3), demonstrating considerable variability in infants entering their first RSV season. As expected, levels decreased with increased age at randomization, with infants older than 6 months having the lowest RSV antibody levels. This finding may explain why older infants are still at risk for severe RSV disease as 10.8% and 6.1% of infants greater than 6 months of age in the placebo group had a medically attended RSV LRTI in the phase 2b and MELODY studies^15,21^. This finding is in agreement with a systematic analysis performed by Li et al. which reported global hospitalization rates and hospital case fatality rates that ranged from 7.4% to 14.3% and from 0.4% to 1.2%, respectively, in infants 6–12 months of age^2^. Of note, approximately 25% of term infants had unmeasurable NAb levels at baseline (Fig. 3), leaving them particularly susceptible to RSV infection during their first RSV season.

We chose to develop nirsevimab as a passive immunization strategy for all infants for four reasons. One, passive immunization overcomes the limitations of gaining an active response to immunization in the immature immune system of neonatal infants; two, the use of palivizumab, an F-specific monoclonal antibody that binds pre- and post-F, has been shown to be safe and effective at preventing RSV disease in preterm infants^27^; three, targeting the pre-F form of F with a NAb potentially minimizes the risk of antibody-dependent enhanced disease associated with non-neutralizing antibodies^28^; and, four, the extended half-life of nirsevimab enables protection for infants across an entire RSV season following a single i.m. administration.

The estimated half-life of maternal RSV antibodies in the phase 2b and MELODY studies was found to be similar to that of previous studies (36–38 days)^12,25^, which is longer than the standard IgG1 half-life of approximately 21–28 days (ref. ^29^). However, by 3 months into the first RSV season, RSV NAb levels typically decrease to the point where even in infants with high baseline NAb levels, they are close to unmeasurable, meaning that all infants are susceptible to RSV infection during their first season. In addition, due to the extended half-life, nirsevimab demonstrated a 68.7-day (s.d. 10.9 days) half-life in the MELODY study^15^. Following a single i.m. administration of nirsevimab, by day 31 RSV NAb levels were more than 140-fold higher than baseline and levels remained >50 times higher than baseline at 151 days. Indeed, at day 361, RSV NAb levels remained on average >7 times higher than baseline levels in both the phase 2b and MELODY studies, providing support for the observed protective effect of nirsevimab beyond the typical 5-month RSV season^15^.

Characterizing the RSV seroresponse in infants is important for understanding the incidence of RSV exposure in an infant’s first year of life and can be used to determine whether the infants enrolled in the phase 2b and MELODY studies who received nirsevimab had a natural immune response to RSV. RSV post-F antibody levels over time were used to determine seroresponse rates since nirsevimab binds specifically to the site Ø epitope^8,16,17^ on pre-F and does not bind to post-F. Antibody levels to post-F increased in nirsevimab recipients, suggesting that infants with an RSV exposure who did not require medical attention still mounted an immune response in the presence of nirsevimab. Nonetheless, this immune response among nirsevimab recipients would be expected to be lower compared with placebo recipients, along with the reduction in RSV disease observed clinically in nirsevimab compared with placebo recipients^23^. Higher post-F antibody levels were observed in the placebo versus nirsevimab recipients with a diagnostic-confirmed RSV infection, but differences were only statistically significantly different at day 361 (Fig. 5); trends were similar, although not statistically significant, with antibodies against Ga and Gb. While our phase 2b and MELODY clinical trials demonstrate the efficacy of nirsevimab at reducing medically attended and more severe disease forms (including those requiring hospitalization), the presence of natural immune responses to RSV remaining balanced between treatment groups suggests that RSV exposure in nirsevimab-immunized infants was accompanied by subclinical manifestations of disease, indicating that sterilizing immunity is not induced by nirsevimab. Interestingly, in breakthrough LRTI cases in the nirsevimab group, viral load was not decreased as compared with placebo. It may be hypothesized that nirsevimab can exert greater impact on viral replication before lower respiratory tract involvement, potentially blunting the infection in the upper airways. However, viral load data were only available at a single timepoint and may be confounded by the ability to study viral kinetics in breakthrough infections through area under the curve data.

We chose not to use the conventional vaccine definition of ≥4-fold increase from baseline as a definition for seroresponse^30^ because it underestimates the number of infants exposed to RSV, since it does not take into consideration maternal antibody levels at birth and subsequent decay. Based on the calculated 36–38-day maternal antibody half-life, an infant’s RSV-specific antibody levels should decrease by more than 1,000-fold over 10 half-lives (2^10^) throughout the course of a 361-day study. Using a ≥4-fold increase from baseline criteria underestimated an RSV exposure as it identified only 46% of infants who had a medically attended RSV LRTI as having a seroresponse. Therefore, we used a statistically based cut-off of >0.07-fold-change at day 151 or >0.02 at day 361 in RSV post-F-specific antibody levels as our criteria for seroresponse (Supplementary Information Section 1). Based on these criteria, 94% and 95% of placebo and nirsevimab recipients, respectively, in the phase 2b study and 95% and 100%, respectively, in MELODY had a seroresponse following a diagnostic-confirmed RSV infection. In addition, 70% and 69% of the placebo and nirsevimab recipients who did not have a diagnostic-confirmed RSV infection had a seroresponse in the phase 2b study and 63% and 68% of the placebo and nirsevimab recipients who did not have a diagnostic-confirmed RSV infection had a seroresponse in the MELODY study, respectively. These rates are similar to the 69% of infants exposed to RSV in their first year of life reported by Glezen et al. in 1986, suggesting that the overall levels of exposure to RSV in the first year of life have remained constant^31^. Specifically, the neutralizing RSV antibody levels in nirsevimab recipients with undetectable nirsevimab at day 361 who had a diagnostic-confirmed RSV infection were similar to those in placebo recipients with a confirmed RSV infection, suggesting that nirsevimab recipients mounted a NAb response following infection (Fig. 6c). Importantly, there was no evidence of enhanced disease in these nirsevimab recipients and there was even a trend towards a reduction in non-RSV respiratory tract infections in nirsevimab versus placebo recipients^15^. Of note, previous studies did not find evidence of increased risk of severe RSV infection after administration of palivizumab^32^. These data also demonstrate that, akin to maternal antibodies, nirsevimab provides protection against disease, but without sterilizing immunity therefore still allows the stimulation of the immune system to generate an active response, including NAbs. Further studies on natural NAb responses, including profiling the antibody repertoire to specific antigenic sites at which NAbs are targeted (that is, fusion protein site Ø versus antigenic sites I–V), will be part of future investigations.

The strengths of our analyses are the large and diverse populations from two complementary randomized, double-blind, placebo-controlled studies, one in preterm infants and one in late preterm and full-term infants. The two studies were enrolled over 4 yr in the Northern and Southern Hemispheres and included both RSV A and RSV B subtypes. The geographically and ethnically diverse populations included over 2,000 infants across the two studies, with several hundred samples available at different timepoints. Both studies followed the placebo- and nirsevimab-treated infants for 1 yr, allowing characterization of the immune response to RSV based on chronological age, gestational age, sex and hemisphere, making this one of the largest studies to characterize the magnitude and kinetics of an RSV immune response in infants under 1 yr of age.

Limitations of this study included: (1) Not every infant had consent for sample use or had a sample available for testing. (2) Infants from the phase 2b study were less diverse geographically due to restrictions related to future use consent laws for biosamples in several countries. (3) The COVID-19 pandemic created an off-cycle RSV season in 2020–2021 where lockdowns, masking and social distancing changed the incidence and prevalence of RSV. (4) The first timepoint to collect serum in the phase 2b study was at day 91 and the first timepoint in the MELODY study was day 31, making it difficult to compare RSV NAb levels between the two studies during the first 3 months post-dose (the day 151 and 361 samplings, however, were harmonized across the studies, enabling comparison). (5) Infants ≥5 kg in the phase 2b study received nirsevimab 50 mg, whereas infants ≥5 kg in the MELODY study received 100 mg, in line with the weight-banded dosing regimen. This difference in dosing may explain the lower RSV NAb levels seen at day 151 and day 361 between the phase 2b study versus MELODY. (6) It was not possible to distinguish between host RSV NAb levels versus nirsevimab levels post administration of nirsevimab and future studies are planned to measure the proportion of the RSV NAb levels that target the pre-F form of F following an RSV exposure in placebo versus nirsevimab recipients. (7) Some infants aged >6 months at the time of enrollment appeared to have already been exposed to RSV based on RSV antibody levels. To mitigate the impact of high antibody levels in our half-life estimation, data strongly indicating previous RSV exposure in infants >4.5 months were excluded from the calculation of maternal pre-F and post-F antibody half-life. A large proportion of baseline NAb samples were below the lower limit of quantification (LLOQ) in older infants, affecting the half-life estimation. To avoid bias in the estimation of NAb half-life, data from infants ≥4 months of age were excluded. (8) Given the highly potent nature of nirsevimab, it is possible that ‘undetectable levels’ of nirsevimab may represent either the absence of nirsevimab or the lower boundary of the sensitivity of the assay used for detection. If the latter is true, then assays may overestimate endogenous RSV NAb levels; future studies will investigate this possibility.

In conclusion, the results from the phase 2b and MELODY studies in preterm, late preterm and full-term infants show that nirsevimab provided sustained, high levels of RSV NAb throughout the first RSV season when baseline maternal antibodies were waning, and most nirsevimab recipients still had higher RSV NAb levels than placebo recipients after 1 yr. Importantly, during a crucial period when infants’ immune systems are still developing, nirsevimab prevented RSV disease while allowing the development of an immune response to RSV.

## Methods

### Clinical protocols

MELODY was a phase 3, double-blind, randomized, placebo-controlled trial in term and late preterm infants (gestational age of ≥35 weeks) that started on 23 July 2019 and is ongoing (NCT03979313; see the Data availability statement for access to the MELODY protocol). The phase 2b study was a randomized, placebo-controlled trial in preterm and late preterm infants (gestational age of ≥29 to <35 weeks) that started on 3 November 2016 and completed on 6 December 2018 (NCT02878330; protocol available at Clinicaltrials.gov). Together, the phase 2b and MELODY studies enrolled infants (male and female) across 4 yr in both the Northern and Southern Hemispheres: the phase 2b study was performed at 164 sites in 23 countries; MELODY was performed in 160 sites in 21 countries^15,21^. In both studies, infants without a previous RSV infection were randomized 2:1 to receive a single i.m. injection of nirsevimab (phase 2b: all infants received 50 mg; MELODY: infants weighing <5 kg received 50 mg, infants weighing ≥5 kg received 100 mg) or placebo before their first RSV season. Primary and secondary endpoints included the occurrence of medically attended RSV-associated LRTI and RSV-associated hospitalization up to 150 days post-dose, respectively. Although data are reported by gestational age, infants could be any age before their first RSV season at study entry. Consent was obtained for both analyses before the studies were initiated; for MELODY, this was specifically for this antibody analysis; for phase 2b, consent was obtained for future use of samples.

Serum samples were collected at predetermined post-dose timepoints based on availability (phase 2b: baseline and days 91, 151 and 361; MELODY: baseline and days 31, 151 and 361) and were kept frozen at −80 ±10 °C before analysis. Analyses were performed to determine antibody concentrations to RSV in infants receiving placebo or nirsevimab with and without a diagnosed RSV infection; all RSV antibodies measured at baseline (pre-dose) were assumed to be maternal antibodies.

The ability to measure antibodies against individual RSV proteins is integral to the analysis of immune responses to RSV. In the presence of nirsevimab, a pre-F protein NAb, it is difficult to distinguish the nirsevimab contribution of pre-F NAb levels from the infant’s own humoral adaptive immune response to an RSV infection and from maternally transferred antibodies. RSV post-F antibody levels, along with Ga, Gb and N IgG antibodies, are the best indicators of maternal RSV antibodies and/or the infant’s own immune response to RSV in the presence of nirsevimab; methods to quantify these specific antibodies are well established^33–39^.

### RSV microneutralization assay

An RSV microneutralization assay was used to measure NAb concentration^40^. Serum samples were heat-inactivated and then preincubated for 1 h with a known quantity of a recombinant RSV A that expressed green fluorescent protein (GFP) (Aragen BioSciences, Inc, lot no. PC-071-014). Subsequently, the sera/virus mixture was incubated with Vero cells for 22–24 h. Viral infection was determined by counting the number of GFP-positive cells (fluorescent foci units (FFU)) using a cell imaging reader. NAb concentrations were determined by interpolating the FFU response from the serially diluted pooled serum reference standard curve calibrated to the World Health Organization (WHO) 1st International Standard for Antiserum to RSV—National Institute for Biological Standards and Control, code 16/284 (ref. ^41^), and reported in IU ml^−1^ (PPD Vaccines, Richmond, VA, USA). The LLOQ for the anti-RSV neutralization assay was 50 IU ml^−1^.

### Multiplex RSV serology IgG assay

A multiplex RSV serology assay was used to determine RSV-specific IgG antibody concentrations using an indirect binding format, and was performed at PPD Vaccines, Richmond, VA, USA^38^. Briefly, the serum reference calibration curve, quality-control serum samples and test samples were incubated on a 96-well, Multiplex Custom RSV Serology SECTOR plate coated with RSV antigens (pre-F, post-F, Ga, Gb and N) provided to Meso Scale Discovery (MSD) by AstraZeneca^38,42^. Pre-F protein (DSCav-1) was manufactured under license from the National Institutes of Health (License Application Number A-061-2018). Anti-RSV antibodies present in serum samples were bound to the plates to form an antibody–antigen complex. Subsequently, a monoclonal SULFO-TAG-labeled anti-human specific IgG antibody (MSD, lot no. W0019421-20191211-WTK) was used to bind to the serum antibodies. The resulting electrochemiluminescence was measured in relative light units using an MSD SECTOR S600 plate reader. Test sample antibody concentrations were determined by interpolating their electrochemiluminescence response from the standard curve generated from the serially diluted pooled serum reference standard. Antigen-specific antibody concentrations were reported in arbitrary units per milliliter (AU ml^−1^)^42^. The assay was qualified before phase 2b testing and then validated before testing samples from MELODY. The LLOQs for RSV IgG antibodies established during qualification were pre-F 696 AU ml^−1^; post-F 165 AU ml^−1^; Ga 81 AU ml^−1^; Gb 90 AU ml^−1^; and N 20 AU ml^−1^. In the validated assay, the LLOQs were re-established at pre-F 62 AU ml^−1^; post-F 41 AU ml^−1^; Ga 193 AU ml^−1^; Gb 145 AU ml^−1^; and N 34 AU ml^−1^.

### Statistical analyses

For all measurements, including NAb, pre-F, post-F, Ga, Gb or N, the GMC and the GMFR from baseline were determined at each prespecified timepoint by treatment group. GMCs and corresponding 95% CIs were summarized by treatment group. For GMFR calculations, only infants with both baseline and post-baseline results were included in the analysis. The 95% CIs of GMC and GMFR were calculated assuming log normal distribution. For all calculations, measurements of antibody levels less than the LLOQ were imputed at half the LLOQ.

### Estimation of antibody half-life

Maternal antibody half-life was calculated based on the assumption that no infant was exposed to RSV before enrollment. RSV antibody half-life was estimated in both phase 2 and MELODY studies, based on pooled baseline data from infants, using noncompartmental methods (log-linear regression) assuming a mono-exponential decay. Data below the LLOQ were imputed to half the LLOQ.

Data exclusions were made to avoid bias from likely RSV exposure and data below the LLOQ. For additional details see Supplementary Information Section 2.

### Determination of seroresponse cut point

To measure RSV exposure, seroresponse cut points were determined via a statistical method using RSV post-F antibody levels measured from the serum samples (Supplementary Information Section 1). Diagnostic-confirmed RSV-positive infants were used to define the true RSV positives to establish the cut point. For each infant, the antibody concentration fold-change from baseline was calculated for day 151 and/or day 361. Cut points were based on tolerance limits, and taking into account maternal antibody decay, were calculated to be 0.07- and 0.02-fold-change from baseline for day 151 and day 361, respectively. An infant was defined as seropositive (exposed to RSV) if the antibody fold-change was above the respective cut point (>0.07 at day 151 or >0.02 at day 361) for at least one timepoint.

The analysis was limited to infants that had a baseline RSV post-F antibody result and either day 151 or day 361 result available.

### Inclusion and ethics

The trials from which these data were gathered were performed in accordance with the principles of the Declaration of Helsinki and the International Council for Harmonisation Good Clinical Practice guidelines. Each site had approval from an institutional ethics review board or ethics committee, and appropriate written informed consent was obtained for each participant. Data were collected by clinical investigators and analyzed by ClinChoice (a contract research organization).

### Reporting summary

Further information on research design is available in the Nature Portfolio Reporting Summary linked to this article.

## Online content

Any methods, additional references, Nature Portfolio reporting summaries, source data, extended data, supplementary information, acknowledgements, peer review information; details of author contributions and competing interests; and statements of data and code availability are available at 10.1038/s41591-023-02316-5.

### Supplementary information

Supplementary Information Supplementary Information Section 1 Seroresponse cut-point analysis, Section 2 Estimation of antibody half-life, Tables 1–5 and Independent Ethics Committees/Institutional Review Boards consulted.

Reporting Summary

## Figures and Tables

**Extended Data Table 1:**
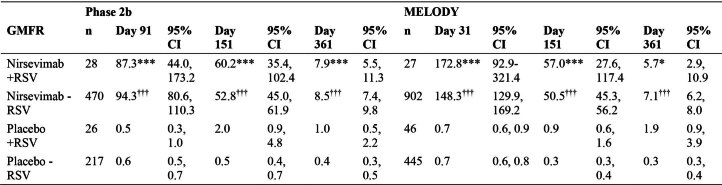
RSV NAb GMFR through day 361 by treatment and medically attended, diagnostic-confirmed RSV infection

***P < 0.001, nirsevimab vs placebo with diagnostic-confirmed RSV; ^†††^*P* < 0.001 nirsevimab vs placebo without diagnostic-confirmed RSV. All were *P* < 0.0001 except for day 361 in MELODY comparing infants with diagnostic-confirmed RSV, which was *P* = 0.0419. n denotes the number of infants who had a serum sample available at day 361. CI, confidence interval; GMFR, geometric mean fold rise; NAb, neutralizing antibody; RSV, respiratory syncytial virus.

**Extended Data Table 2:**
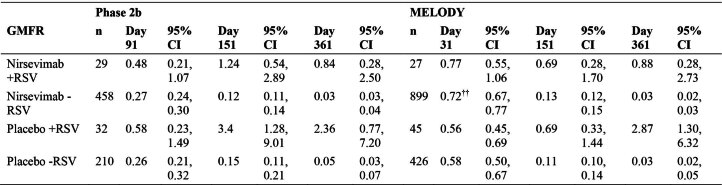
RSV post-F antibody GMFR through day 361 by treatment and medically attended, diagnostic-confirmed RSV infection

^††^*P* < 0.01 nirsevimab vs placebo without diagnostic-confirmed RSV (*P* = 0.0047). CI, confidence interval; F, fusion protein; GMFR, geometric mean fold rise; n, number of infants; RSV, respiratory syncytial virus.CI, confidence interval; F, fusion protein; GMFR, geometric mean fold rise; n, number of infants; RSV, respiratory syncytial virus.

## Data Availability

Data underlying the findings described in this paper may be obtained in accordance with AstraZeneca’s data sharing policy described at https://astrazenecagrouptrials.pharmacm.com/ST/Submission/Disclosure. Data for studies directly listed on Vivli can be requested through Vivli at www.vivli.org. Data for studies not listed on Vivli could be requested through Vivli at https://vivli.org/members/enquiries-about-studies-not-listed-on-the-vivli-platform/. The AstraZeneca Vivli member page is also available, outlining further details: https://vivli.org/ourmember/astrazeneca/.
