# Author Correction: Durability of neutralizing RSV antibodies following nirsevimab administration and elicitation of the natural immune response to RSV infection in infants

**DOI:** 10.1038/s41591-024-03006-6

**Published:** 2024-04-22

**Authors:** Deidre Wilkins, Yuan Yuan, Yue Chang, Anastasia A. Aksyuk, Beatriz Seoane Núñez, Ulrika Wählby-Hamrén, Tianhui Zhang, Michael E. Abram, Amanda Leach, Tonya Villafana, Mark T. Esser

**Affiliations:** 1 Translational Medicine, Vaccines & Immune Therapies, BioPharmaceuticals R&D, AstraZeneca, Gaithersburg, MD USA; 2 Biometrics, Vaccines & Immune Therapies, BioPharmaceuticals R&D, AstraZeneca, Madrid, Spain; 3 Clinical Pharmacology & Quantitative Pharmacology, R&D, AstraZeneca, Gothenburg, Sweden; 4 Data Sciences and Quantitative Biology, R&D, AstraZeneca, Gaithersburg, MD USA; 5 Clinical Development, Vaccines & Immune Therapies, Biopharmaceuticals R&D, AstraZeneca, Gaithersburg, MD USA; 6 Vaccines & Immune Therapies, Biopharmaceuticals R&D, AstraZeneca, Gaithersburg, MD USA

**Keywords:** Viral infection, Vaccines, Immunization

Correction to: *Nature Medicine* 10.1038/s41591-023-02316-5, published online 24 April 2023.

In the version of the article initially published, in the Fig. 6a Phase 2b, Nirsevimab pie chart, “(*n* = 294)” has been corrected from “(*n* = 134)” in the HTML and PDF versions of the article.

