# Supplementary material for: Durability of neutralizing RSV antibodies following nirsevimab administration and elicitation of the natural immune response to RSV infection in infants
Source: Nat Med. 2023 Apr 24;29(5):1172–9. doi: 10.1038/s41591-023-02316-5 (PMC10202809; doi:10.1038/s41591-023-02316-5)
Supplement: Supplementary file 1 — Supplementary Information Section 1 Seroresponse cut-point analysis, Section 2 Estimation of antibody half-life, Tables 1–5 and Independent Ethics Committees/Institutional Review Boards consulted. [file 41591_2023_2316_MOESM1_ESM.pdf]

nature medicine

Article https://doi.org/10.1038/s41591-023-02316-5

Durability of neutralizing RSV antibodies following nirsevimab administration and elicitation of the natural immune response to RSV infection in infants

In the format provided by the authors and unedited

SUPPLEMENTARY INFORMATION

Section 1. Seroresponse cut-point analysis

Sample selection summary. There were 117 diagnostic-confirmed RSV positive infants with LRTI before Day 361 with available RSV post-F antibody data (53 infants from Phase 2b and 64 infants from MELODY from both treatment groups). All 117 RSV-positive infants who had a baseline sample, as well as either Day 151 and/or day 361 sample were included in the infant-level assessment analysis. Out of 117 RSV-positive infants, 91 infants had both day 151 and day 361 samples, 11 infants had Day 151 but not Day 361, and 15 had Day 361 and not Day 151.

Seroresponse cut points were defined using RSV post-F antibody levels fold change over baseline in 93 RSV diagnostic-confirmed RSV positive infants with LRTI before Day 151 (49 infants from Phase 2b and 44 infants from MELODY). Since RSV post-F antibody levels continue to decline after Day 151, cut points for seroresponse were determined for Day 151 and Day 361 separately.

Statistical method. Cut point can be defined using beta-expectation tolerance limit (Shen et al 2015). By definition, a p% tolerance limit means (1-p)% of the future samples in this category would fall above this value. For example, a lower 5% tolerance limit-based cut point to identify a seroconverted sample set will identify approximately 95% RSV-positive samples.
A linear model was fitted to the natural log-transformed fold change data on day 151 and day 361 separately:

$$y_i = \mu + e_I \qquad (1)$$

where y_i is the ln fold change from baseline for the ith infants; μ is the mean ln fold change for the RSV-positive population; $e_i \sim N(0, \sigma_e^2)$ is the residual error.

Model (1) was fitted using the Bayesian method. Priors for the parameters were obtained from fitting model (1) to the log-transformed fold-change data using lm() in R:

$\mu \sim N(0.85, 1)$ for day 151; $\mu \sim N(0.36,\ 1)$ for day 361;

$\sigma_e \sim$ half-cauchy with scale 0.310 for both day 151 and day 361.

The lower 1%, 3%, and 5% beta-expectation tolerance limit were obtained from posterior samples.

Seroresponse cut-point values. The cut points were based on 1%, 3%, and 5% tolerance limit for day 151 and day 361 post-F antibody concentration fold change over baseline are shown in Supplementary Table 3.

Seroresponse incidence rate. The infant-level seroresponse was evaluated using cut points based on 1%, 3%, and 5% tolerance limit. Additionally, traditional 4-fold change from baseline definition of seroconversion was also evaluated.

Using 4-fold change as a measure of seroresponse resulted in only 46% clinical sensitivity (true positive rate) for the diagnostic-confirmed RSV-positive infants (Supplementary Table 4). This is expected due to maternal antibody levels waning over time and thus using 4-fold change from baseline to define seroresponse is not ideal for RSV. Using 5% tolerance limit-based cut point resulted in 96% overall clinical sensitivity (true positive rate) for the diagnostic-confirmed RSV-positive infants. Based on the clinical sensitivity data (true positive rate), the 5% tolerance limit cut point was selected as the most appropriate. Clinical specificity (true negative rate) could not be defined because the diagnostic-confirmed RSV-negative sample set was not available since infants can be exposed to RSV and have subclinical symptoms that would not have been medically diagnosed as RSV. Therefore, RSV "not diagnostic-confirmed" infants included both RSV exposed and not exposed infants.

Overall infant-level seroresponse, along with each treatment arm and individual studies, using a 5% tolerance limit cut point and 4-fold change are shown in Supplementary Table 5.

Application. RSV seroresponse was defined using the cut-point analysis, based on RSV post-F measured antibody levels over time from diagnostic-confirmed RSV-positive infants. An infant was defined as seropositive if the fold change in post-F antibody concentration from baseline is above 0.07 on day 151 or is above 0.02 on day 361. Applying the cut-point analysis resulted in improved overall

clinical sensitivity (96%) when compared with traditional 4-fold change over baseline to define seroresponse (46%). RSV exposure based on this definition of seroresponse cut points was applied to each individual study by cohort and by treatment.

Section 2. Estimation of antibody half-life.

An approximate estimate of the half-life of maternal antibody in each study population was achieved by pooling baseline data from all infants and treating as if it originated from the same individual. The half-life was estimated, assuming a mono-exponential decay, by standard noncompartmental analysis using log-linear regression of antibody versus age at randomization. The half-life is determined as $-\ln(2)/k$ where k is the slope of the linear regression of ln(Antibody) vs age, and ln is the natural logarithm.

Data below the LLOQ was set to half the LLOQ; 25 IU/ml, for RSV NAb levels, 348 and 82.5 for pre- and post-F in Study 3, respectively, and 31 and 20.5 for pre- and post-F in MELODY, respectively. NAb data from infants ≥4 months of age were excluded due to the high percentage of samples below the LLOQ (>32%). Pre-F and post-F data strongly indicated exposure to RSV (and antibody levels were therefore not maternal) and were thus, excluded from the half-life calculation (>4.5 months + RSV pre-/post-F levels >100,000 IU/ml and >7.5 months + RSV pre-/post-F levels >10,000 IU/ml). Sensitivity analyses including all pre- and post-F data were performed and resulted in increased point estimates of half-lives with generally larger confidence intervals (95%CI); Study 3 pre-F 40 (38-43) days, Study 3 post-F 41 (39-44) days, MELODY pre-F 44 (42-46) days, MELODY post-F 45 (43-48) days.

Supplementary Table 1. Baseline demographics and participant characteristics p-values to be added

Variable	Phase 2b†			MELODY†		
	**Nirsevimab (n = 498)**	**Placebo (n = 243)**	**Full cohort (n = 1453)**	**Nirsevimab (n = 929)**	**Placebo (n = 473)**	**Full primary cohort (n = 1490)**
Gestational age group						
<31 weeks	34 (6.8)	19 (7.8)	140 (9.6)	–	–	-
≥31 to <33 weeks	121 (24.3)	66 (27.2)	408 (28.1)	–	–	-
≥33 to <35 weeks	343 (68.9)	158 (65.0)	905 (62.3)	–	–	-
≥35 to <37 weeks	–	–	-	125 (13.5)	74 (15.6)	208 (14.0)
≥37 weeks	–	–	-	804 (86.5)	399 (84.4)	1280 (86.0)
Age at randomization, mean (range)	3.3 (0.1-11.9)	3.4 (0.2-9.5)	3.4 (0.1-11.9)	2.9 (0.0-11.1)	3.0 (0.0-11.0)	3.0 (0.0-11.1)
≤3 months	262 (52.6)	119 (49.0)	773 (53.2)	535 (57.6)	271 (57.3)	862 (57.9)
>3 to ≤6 months	166 (33.3)	87 (35.8)	473 (32.6)	301 (32.4)	158 (33.4)	479 (32.1)
>6 months	70 (14.1)	37 (15.2)	207 (14.2)	93 (10.0)	44 (9.3)	149 (10.0)
Sex						
Female	235 (47.2)	100 (41.2)	692 (47.6)	431 (46.4)	245 (51.8)	721 (48.4)
Male	263 (52.8)	143 (58.8)	761 (52.4)	498 (53.6)	228 (48.2)	769 (51.6)
Ancestry‡						
American Indian or Alaska Native	0 (0.0)	0 (0.0)	1 (0.1)	57 (6.1)	26 (5.5)	83 (5.6)
Asian	5 (1.0)	5 (2.1)	15 (1.0)	35 (3.8)	18 (3.8)	54 (3.6)
Black or African American	41 (8.2)	10 (4.1)	256 (17.6)	271 (29.2)	134 (28.3)	422 (28.4)
Native Hawaiian or other Pacific Islander	3 (0.6)	0 (0.0)	11 (0.8)	6 (0.6)	4 (0.8)	11 (0.7)
White	438 (88.0)	218 (89.7)	1048 (72.1)	484 (52.2)	256 (54.1)	796 (53.5)
Other	10 (4.1)	11 (2.2)	121 (8.3)	74 (8.0)	35 (7.4)	121 (8.1)
Weight group						
<5 kg	294 (59.0)	131 (54.1)	860 (59.5)	371 (39.9)	183 (38.7)	595 (40.0)
≥5 kg	204 (41.0)	111 (45.9)	585 (40.5)	558 (60.1)	290 (61.3)	893 (60.0)
Hemisphere						
Northern	434 (87.1)	216 (88.9)	988 (68.0)	630 (67.8)	322 (68.1)	1028 (69.0)
Southern	64 (12.9)	27 (11.1)	465 (32.0)	299 (32.2)	151 (31.9)	462 (31.0)

Data reported as n (%) unless otherwise stated. There were no statistically significant differences between nirsevimab and placebo recipients in terms of demographics and participant characteristics.

†Sampling was pre-specified in MELODY; in the phase 2b study, sampling was only performed in infants where appropriate consent had been provided. *n* denotes population with ≥1 sample available for testing at any time point.

‡The population included from the phase 2b study was less diverse with the exclusion of South Africa due to future use consent laws.

n, number of infants.

Supplementary Table 2. Baseline RSV-specific NAb levels by study

	Phase 2b	MELODY	p-value
NAb			
n	741	1402	
GMC, IU/ml (95% CI)	86.77 (79.34, 94.90)	134.07 (125.28, 143.48)	<0.0001
Pre-F			
n	722	1396	
GMC, AU/ml (95% CI)	17769.15 (15871.95, 19893.13)	29603.04 (27472.38, 31898.94)	<0.0001
Post-F			
n	729	1397	
GMC, AU/ml (95% CI)	8383.93 (7509.99, 9359.57)	12878.69 (11932.08, 13900.41)	<0.0001
Ga			
N	727	1397	
GMC, AU/ml (95% CI)	1694.13 (1455.66, 1857.01)	2488.92 (2294.69, 2699.60)	<0.0001
Gb			
n	729	1390	
GMC, AU/ml (95% CI)	4152.68 (3698.76, 4662.30)	6438.91 (5932.73, 6988.29)	<0.0001
N			
n	729	1392	
GMC, AU/ml (95% CI)	777.09 (713.61, 846.21)	1138.35 (1059.73, 1222.81)	<0.0001

Two-sided p-values were calculated based on the F statistic from ANOVA, without adjustment. All p-values were as written.

AU/ml, arbitrary units per milliliter; CI, confidence interval; F, fusion protein; Ga, attachment protein G RSV subtype A; Gb, attachment protein G subtype B; GMC, geometric mean concentration; IU/ml, International Units per milliliter; n, number of infants; N, nucleocapsid N; NAb, neutralizing antibodies; RSV, respiratory syncytial virus.

Supplementary Table 3. Cut-point values for day 151 and day 361 (post-F fold change over baseline)

Tolerance limit	Day 151	Day 361
5%	0.07	0.02
3%	0.04	0.01
1%	0.01	0.004

F, fusion protein.

Supplementary Table 4. Clinical sensitivity (true positive rate) for different tolerance limit cut points for day 151 or day 361 post-F IgG antibody

RSV	1%, n (%)	3%, n (%)	5%, n (%)	4-fold, n (%)
Diagnostic-confirmed positive (*n* = 117)	117 (100)	115 (98)	112 (96)	54 (46)
Not diagnostic-confirmed (*n* = 1,841)	1,799 (98%)	1,604 (87)	1,240 (67)	197 (11)

Infants were defined as seropositive if the antibody fold-change from baseline was above the respective cut point (>0.07 at day 151 or >0.02 at day 361) for at least one time point (see Supplementary Information Section 1.).

Of the 117 diagnostic-confirmed positive cases, 53 infants were from the phase 2b study and 64 infants were from the MELODY study.

F, fusion protein; IgG, immunoglobulin G; n, number of infants; RSV, respiratory syncytial virus.

Supplementary Table 5. Analysis of seroresponse comparing incidence of diagnostic-confirmed RSV and not diagnostic-confirmed infants across treatments and studies

	Tolerance limit, n (%)		Fold-change, n (%)	
	>5%	<5%	>4-fold	<4-fold
Overall total	1,352 (69)	606 (31)	251 (13)	1,707 (87)
Diagnostic-confirmed RSV (n = 117)	112 (96)	5 (4)	54 (46)	63 (54)
Without diagnostic-confirmed RSV (n = 1,841)	1,240 (67)	601 (33)	197 (11)	1,644 (89)
Overall nirsevimab treated	895 (69)	394 (31)	128 (10)	1,161 (90)
Diagnostic-confirmed RSV (n = 46)	45 (98)	1 (2)	14 (30)	32 (70)
Without diagnostic-confirmed RSV (n = 1,243)	850 (68)	393 (32)	114 (9)	1,129 (91)
Overall placebo treated	457 (68)	212 (32)	123 (18)	546 (82)
Diagnostic-confirmed RSV (n = 71)	67 (94)	4 (6)	40 (56)	31 (44)
Without diagnostic-confirmed RSV (n = 598)	390 (65)	208 (35)	83 (14)	515 (86)
Phase 2b nirsevimab treated	315 (70)	135 (30)	61 (14)	389 (86)
Diagnostic-confirmed RSV (n = 22)	21 (95)	1 (5)	8 (38)	14 (62)
Without diagnostic-confirmed RSV (n = 428)	294 (69)	134 (31)	53 (12)	375 (88)
Phase 2b placebo treated	172 (73)	63 (27)	48 (20)	187 (80)
Diagnostic-confirmed RSV (n = 31)	29 (94)	2 (6)	17 (54)	14 (46)
Without diagnostic-confirmed (n = 204)	143 (70)	61 (30)	31 (15)	173 (85)
MELODY nirsevimab treated	580 (69)	259 (31)	67 (8)	772 (92)

Diagnostic-confirmed RSV (n = 24)	24 (100)	0 (0)	6 (25)	18 (75)
Without diagnostic-confirmed RSV (n = 815)	556 (68)	259 (32)	61 (7)	754 (93)
MELODY placebo treated	285 (66)	149 (34)	75 (17)	359 (83)
Diagnostic-confirmed RSV (n = 40)	38 (95)	2 (5)	23 (58)	17 (42)
Without diagnostic-confirmed RSV (n = 394)	247 (63)	147 (37)	52 (13)	342 (87)

Infants were defined as seropositive if the antibody fold-change from baseline was above the respective cut point (>0.07 at day 151 or >0.02 at day 361) for at least one time point (see Supplementary Information Section 1.). All measured RSV post-F values were used in this comparison including values imputed at ½ the LLOQ.

n, number of infants; RSV, respiratory syncytial virus.

Independent Ethics Committees/Institutional Review Boards consulted

Site Number	Name/Address of IRB/IEC
**Phase 2b**	
2002923	Pharma Ethics 123 Amcor Road Lyttelton Manor Centurion Pretoria Gauteng
2003359	MetroHealth Medical Center IRB 2500 MetroHealth Dr. Rammelkamp Bldg. Room 103 Cleveland Ohio
2002934	CEP Investiga - Instituto de Pesquisas Avenida Romeu Tortima, 739 - Cidade Universitária Campinas Sao Paulo
2002970, 2003091, 2003395, 2003007, 2003356, 2003405, 2003355, 2003354, 2003004, 2003353, 2002971, 2003124, 2003350, 2003394, 2003092, 2003348, 2003078, 2002974, 2003036, 2003346, 2003340, 2003441, 2003167, 2003338, 2003337, 2003442, 2003068, 2003038, 2003342, 2003335, 2003399, 2003086, 2003444, 2003400, 2003332, 2002976, 2003402, 2003347, 2003403, 2003125, 2003329, 2003336, 2003079, 2003401, 2003407	Copernicus Group IRB 5000 CentreGreen Way Suite 200 Cary North Carolina Adams, Gregory
2002935	CEP da Universidade Federal de Minas Gerais Avenida Presidente Antonio Carlos 6627 Unidade Administrativa II Belo Horizonte Minas Gerais Andrade
2002947	CEIC de Galicia C/ San Lázaro, s/n Secretaria Xeral. Conselleria de SanidadeDirección Santiago de Compostela La Coruña Ares
2002948	CEIC de Galicia C/ San Lázaro, s/n Secretaria Xeral. Conselleria de SanidadeDirección Santiago de Compostela La Coruña Arimany Montaña,
2003358	Medical University of South Carolina IRB 19 Hagood Avenue 6th floor, Suite 601 Charleston South Carolina
2002918	Wits Health Consortium 31 Princess of Wales Terrace Parktown Johannesburg Gauteng
2002998	Comite Etico Cientifico del Servicio de Salud Metropolitano Sur Santa Rosa 3453, Piso 1 San Miguel Santiago
2003034	CESC della Provincia di Padova Presso Azienda Ospedaliera di Padova_Via Giustiniani 1 Padova
2002939	CEP da Faculdade de Ciências Médicas e da Saúde de Juiz de Fora SUPREMA/MG Alameda Salvaterra, 200 Bairro Salvaterra Juiz de Fora Minas Gerais Bastos
2003060	CEP da Faculdade de Medicina de Botucatu - UNESP/SP Distrito de Rubião Junior Botucatu Sao Paulo

Site Number	Name/Address of IRB/IEC
2003000	Comitato Etico per la Sperimentazione Clinica delle Provincie di Verona e Rovigo P.le Stefani, 1 Verona
2002919	Pharma Ethics 123 Amcor Road Lyttelton Manor Centurion Pretoria
2002910	Monash Health Human Research Ethics Committee (RGO) Level 2, I Block Clayton Victoria
2002953	Comité Etico Científico Servicio de Salud Valdivia Maipú 550, oficina 307 Valdivia
2002920	University of Stellenbosch Ethics Committee Faculty of Health Sciences Francie van Zijl Drive Tygerberg Cape Town Western Cape
2002956	Comité Ético Científico Servicio de Salud Metropolitano Central Victoria Subercaseaux 381, piso 4 Santiago
2003352	UTHSC IRB Office 910 Madison Suite 600 Memphis Tennessee
2003118	Memorial Health Services Research Council 2801 Atlantic Avenue Attn Research Administration Long Beach California
2002972	SUNY IRB 750 East Adams Street CWB 218G Syracuse New York
2003277	McGill University Health Center-Research Ethics Board 2155 Guy Street 2nd Floor, Room 231 Montreal Quebec
2002921	Pharma Ethics 123 Amcor Road Lyttelton Manor Centurion Pretoria Gauteng
2002973, 2003061, 2003069, 2003093, 2003331, 2003447	WIRB 1019 39th Avenue SE Suite 120 Puyallup Washington
2002905	Comité de Ética en Investigación Científica. Hospital Pediátrico Dr. Humberto Notti Bandera de Los Andes 2603 Villa Nueva Guaymallén Mendoza
2002967	R&D University Hospital Southampton NHS Foundation Trust Tremona Road, Level E, Laboratory & Pathology Block, SCBR - MP 138 Southampton Hampshire
2003320	R&D - Brighton and Sussex University Hospitals Royal Sussex County Hospital Level 5 Thomas Kemp Tower Eastern Road Brighton East Sussex
2003065	Azienda Ospedaliera Città della Salute e della Scienza di Torino Corso Bramante 88/90. Torino
2002940	Comitê de Ética em Pesquisa em Seres Humanos do Instituto de Medicina Integral Professor Fernando F Rua dos Coelhos, 300 - Boa Vista Recife Pernambuco Gomes
2002922	University of Cape Town HREC Faculty of Health Sciences Research EC E52-24 Old Main Building Groote Schuur Hospital, Observatory Cape Town Western Cape
2002941	CEP da Universidade Luterana do Brasil - ULBRA Farroupilha, 8001 - Prédio 14 - Sala 224 Bairro São José Canoas Rio Grande do Sul
2003319	R&D - Alder Hey Children's NHS Foundation Trust Eaton Road Liverpool Merseyside
2002968	R&D South West London and St George's Mental Health NHS Trust Department of Mental Health, St George's, University of London, 6th Floor, Hunter Wing, Cranmer Terrace London Greater London

Site Number	Name/Address of IRB/IEC
2002924	Eticka komise IKEM a FTNsP Videnska 800 Praha 4 - Krc
2003343	Sharp Healthcare IRB 7930 Frost St Suite 300 San Diego California
2003341	Winthrop-University Hospital IRB 222 Station Plaza North Suite 521 Mineola New York
2002943	Comitê de Ética em Pesquisa em Seres Humanos do Hospital Pequeno Príncipe Rua Desembargador Motta, 1070 6º andar, sala do NUPE Curitiba Paraná
2003339	Marshall University Office of Research Integrity One John Marshall Drive Huntington West Virginia
2002937	Wits Health Consortium 31 Princess of Wales Terrace Parktown Johannesburg Gauteng
2002950	CEIC de Galicia C/ San Lázaro, s/n Secretaria Xeral. Conselleria de SanidadeDirección Santiago de Compostela La Coruña Martinon
2002944	CEP da Universidade de Passo Fundo/RS Universidade de Passo Fundo - BR 285, Bairro São José Passo Fundo Rio Grande do Sul
2003011	Ann & Robert H. Lurie Children’s Hospital of Chicago Institutional Review Board 225 E. Chicago Avenue Box 59 Chicago Illinois
2003334, 2002975	Chesapeake IRB 7063 Columbia Gateway Drive Suite 110 Columbia Maryland
2003067	Cincinnati Children's Hospital Medical Center IRB 3333 Burnet Ave. MLC 5020 Cincinnati Ohio
2002954	Comité Ético-Científico Servicio de Salud Metropolitano Sur Oriente Av Concha y Toro 3459 Puente Alto Santiago
2003333	Childrens Hospital of Los Angeles-Committee on Clinical Investigations IRB 4650 Sunset Blvd Mail Stop #23 Dr. Andreas Reiff Los Angeles California
2003280	McGill University Health Center-Research Ethics Board 2155 Guy Street 2nd Floor, Room 231 Montreal Quebec
2003005	University of Texas at San Antonio IRB One UTSA Circle MS 4.01.82 San Antonio Texas
2002951	CEIC de Galicia C/ San Lázaro, s/n Secretaria Xeral. Conselleria de SanidadeDirección Santiago de Compostela La Coruña
2002911	Royal Children's Health Services Human Research Ethics Committee (RGO) 50 Flemington Road Parkville Victoria
2002938	Pharma Ethics 123 Amcor Road Lyttelton Manor Centurion Pretoria Gauteng
2003257	Comité Ético-Científico Servicio de Salud Viña del Mar-Quillota Calle Limache #1307 Esquina Peñablanca 2º Piso Viña del Mar Quilodran
2003035	Comitato Etico Regionale della Liguria Largo Rosanna Benzi 10 Farmacia Ospedaliera Genova
2002912	Princess Margaret Hospital for Children Ethics Committee Princess Margaret Hospital Entrance No 6, Hamilton Street Subiaco Western Australia

Site Number	Name/Address of IRB/IEC
2003330	Arnold Palmer Medical Center Institutional Review Board 1401 Kuhl Avenue MP #21 Research Department Orlando Florida
2003328	University of Nebraska Medical Center IRB 987830 Nebaska Medical Center Omaha Nebraska
2002969	R&D - CRN Thames Valley and South Midlands 1st Floor, Manor House The John Radcliffe Hospital, Headley Way Headington Oxford Oxfordshire
2002926	Eticka komise Ustav pro peci o matku a dite Podolske nabrezi 157/36 Praha 4 - Podoli
2002909	Comité Hospitalario de Etica Necochea 675 Bahia Blanca Buenos Aires
2003274	Comité d'Ethique du CHU Ambroise Paré Boulevard Kennedy 2 Mons Van
2002955	Comité de Ética de Investigación en Seres Humanos Av. Independencia 1027, Independencia Santiago Vargas
2003009	Creighton University IRB 2500 California Plaza IRB-Biomedical Omaha Nebraska
2002966	R&D University Hospitals Bristol NHS Foundation Trust Education & Research Centre Level 3 Upper Maudlin Street Bristol Avon
2002999	Comite Etico Cientifico del Servicio de Salud Metropolitano Sur Santa Rosa 3453, Piso 1 San Miguel Santiago Villena
2002927	Eticka komise Nemocnice Havlickuv Brod Husova 2624 Havlickuv Brod Weberova,
2003448	Oklahoma University Health Sciences Center 1105 North Stonewall Avenue Oklahoma City Oklahoma
2003406	Connecticut Children's Medical Center IRB 282 Washington Street. Suite 2 K. Hartford Connecticut
2002946	University of Cape Town HREC Faculty of Health Sciences Research EC E52-24 Old Main Building Groote Schuur Hospital, Observatory Cape Town Western Cape

Site Number	Name/Address of IRB/IEC
**MELODY**	
2004023, 2004025, 2004027, 2004028, 2004030, 2004031, 2004032, 2004118, 2004236, 2004237, 2004239, 2004240, 2004243, 2004253, 2004255, 2004256, 2004258, 2004259, 2004260, 2004261, 2004263, 2004264, 2004267, 2004268, 2004278, 2004279, 2004280, 2004291, 2004292, 2004293, 2004314, 2004315, 2004316, 2004319, 2004323, 2004340, 2004345, 2004376, 2004386, 2004389, 2004394, 2004409, 2004613, 2004614, 2004615, 2004618, 2004624, 2004634, 2004650, 2004652, 2004656, 2004657, 2004664, 2004677, 2004679, 2004680, 2004690, 2004697, 2004699, 2004700, 2004702, 2004746, 2004873, 2005604, 2005605, 2005606	WCG IRB, 212 Carnegie Center, Suite 301, Princeton, NJ 08540, USA
2004026	The University of Oklahoma, Institutional Review Board for the Protection of Human Subjects, 1105N. Stone wall Avenue, Oklahoma City, OK73117(FWA 007961)
2004029	Nemours Office of Human Subjects Protection, Nemours/Alfred I. duPont Hospital for Children, 1600 Rockland Road, Wilmington, DE 19803
2004036	University of Cape Town Human Research Ethics Committee, DEPARTMENT OF PAEDIATRICS AND CHILD HEALTH, RED CROSS WAR MEMORIAL CHILDREN’S HOSPITAL, KLIPFONTEIN ROAD, RONDEBOSCH, 7700
2004039	Stellenbosch University Human Research Ethics Committee, Stellenbosch University, Private Bag X1, Matieland, 7602, Stellenbosch, South Africa
2004043	Servicio De Salud Metropolitano Sur Oriente Comite Etico-Cientifico, Av. Concha y Toro 3459 – Paradero 30, Vic. Mackenna
2004098	UNIVERSIDAD DE CHILE [University of Chile] – FACULTAD DE MEDICINA, HUMAN RESEARCH ETHICS COMMITTEE, Av. Libertador Bernardo O'Higgins 1058, Santiago de Chile
2004103	Comitato Etico per la Sperimentazione Clinica delle Provincie di Verona e Rovigo,P.le Stefani, 1, Verona,37126
2004111	1 Military Hospital Human Research Ethics Committee, Department of Neurology Private bag X 1026 Thaba Tswane 0143
2004117	Dept of health of Chernivtsi city council, Communal Medical Institution City Clinical Childrens' Hospital, 4 Bukovynska St, Chernivtsi, 58001

Site Number	Name/Address of IRB/IEC
2004132	Independent Ethics Committee for Clinical Pharmacology Trials, Drug and Pharmacology Studies Foundation, LA FUNDACIÓN DE ESTUDIOS FARMACOLOGICOS Y DE MEDICAMENTOS, Pte. J. E. Uriburu 774 1º Piso Ciudad Autónoma de Buenos Aires (C1027AAP), Argentina
2004178	Landesärztekammer Baden-Württemberg, Ethik-Kommission, Liebknechtstr. 33, 70565 Stuttgart
2004182	Ethik-Kommission der Bayerischen Landesärztekammer, Mühlbaurstr.16, D-81677 München
2004185	Ethik-Kommission an der Medizinischen Fakultaet der Universitaet Leipzig,Kaethe-Kollwitz-Strasse 82,Haus: Karl-Sudhoff-Institut Leipzig,04109
2004222	Ege University Ethics Committee,Ege Universitesi Tip Fakultesi,Klinik Arastirmalar Etik Kurulu Izmir,35100
2004227	Ministy of Health of Ukrain, Communal Non-Commercial enterprise Saint Zinaida Children's Clinical Hospital of Sumy City Council, 28 Troiiska st, Sumy, 40022
2004229	Vinnytsia regional Children's Clinical Hospital, 108 Khmelnytske shose st, Vinnytsia, 21000. Medical Ethics Commission
2004233	Universidad Pontificia Bolivariana, Calle 78 B No. 72 A 109
2004238	Institutional Review Board, Ann & Robert H. Lurie Children's Hospital of Chicago, 25 East Chicago Avenue, Chicago, Illinois
2004241	Cincinnati Children’s Hospital Institutional Review Board, 3333 Burnet Avenue | MLC 7040 | Cincinnati, OH 45229
2004281	MetroHealth Institutional Review board, 2500 MetroHealth Drive, Cleveland Ohio 44109
2004294	State Institution Academician O.M Lukyanova Institute of Pediatrics, obstetrics and gynecology of national academy of medical sciences of Ukraine, 8 P.Mayborody str Kyiv, 04050
2004295	CORPORACIÓN CIENTÍFICA PEDIÁTRICA, BIOMEDICAL RESEARCH ETHICS COMMITTEE, Calle 5 B5 No. 37 bis - 28
2004296	Ministerio de Salud,<br>Servicio de Salud Valdivia, Scientific Ethics Committee, V. Pérez Rosales 560 - Edificio Prales - Oficina 307 - Piso 3
2004300	Servicio de Salud Metropolitano Norte,<br>esearch Ethics Committee, 272,Calle Maruri 8380000 Independencia Metropolitana de Santiago
2004304	UNIVERSIDAD CES, Calle 10A No. 22 - 04 El Poblado
2004310	Creighton University office of the provost Research Compliance, 2500 California Plaza Omaha, NE 68178-0001
2004322	Communal Non-Commercial enterprise of Kharkiv Regional Council regional Children's clinical hospital, 5 Ozeryanska st Kharkiv, 61093
2004338	Odesa Regional State administration, department of health, communal enterprise, Odesa regional Children's clinical hospital, 3 Ac Vorobiov st, Odes-31, 65031

Site Number	Name/Address of IRB/IEC
2004341	Medical University of South Carolina, 179 Ashley Ave, Charleston, SC 29425
2004351	MUHC Centre for Applied Ethics, 5100, boul. de Maisonneuve Ouest, 5th floor, Office 576, Montréal, Québec, H4A 3T2
2004359	Ethikkommission der Landesärztekammer Rheinland-Pfalz Deutschhausplatz 3 55116 Mainz
2004365	MUHC Centre for Applied Ethics, 5100, boul. de Maisonneuve Ouest, 5th floor, Office 576, Montréal, Québec, H4A 3T2
2004372	COMITÉ DE ÉTICA EN INVESTIGACIÓN VIT, Calle 24 N° 3-02 este
2004391	University of Nebraska Medical Center, 42nd and Emile Streets, Omaha, NE 68198, 402-559-4000
2004396	COMITATO ETICO DELLA FONDAZIONE POLICLINICO UNIVERSITARIO AGOSTINO GEMELLI IRCCS UNIVERSITÀ CATTOLICA DEL SACRO CUORE
2004400	Research Ethics Committee of the Health Sciences Department of the Universidad del Norte, Apartados Aéreos 1569 - 51820, Km. 5 vía Puerto Colombia
2004404	Federico Gomez Children's hospital of Mexico, National Institute of Health research office
2004616	Stony Brook University, Health Sciences Center Room 031, Stony Brook, NY 11794-8111
2004623	UBC C&W Research Ethics Board A2-141A, 950 West 28th Avenue Vancouver, BC V5Z 4H4
2004626	Soroka University Medical Center, Itzhak Rager Blv. Beer Sheva 8458900
2004632	Japanese Red cross Maebashi Hospital IRB 138-Asakuramachi, Maebashi-Shi Gunma
2004633	Ethics Commission at Communal Institution Dnipro City Children's Clinical Hospital No 5 of Dnipro City Council, 5 ivana Akinfiieva st, Dnipro 49027 Ukraine
2004648	Independent Ethics Committee for Clinical Pharmacology Trials, Drug and Pharmacology Studies Foundation, LA FUNDACIÓN DE ESTUDIOS FARMACOLOGICOS Y DE MEDICAMENTOS, Pte. J. E. Uriburu 774 1º Piso Ciudad Autónoma de Buenos Aires (C1027AAP), Argentina
2004658	Nationwide Children's IRB, Nationwide Children’s Hospital, 700 Childrens Drive, Columbus, OH 43205
2004660	Yokosuka Kyosai Hospital IRB, 1-16 Yonegahamadori, Yokosuka Kanagawa
2004662	The University Of Tennessee, Health Science Centre Institutional Review Board, 910 Madison Avenue, Suite 600, Memphis, TN 38163
2004667,	Conjoint Health Research Ethics Board, Research Services Office, 2500 University Drive, NW, Calgary AB T2N 1N4
2004668	Jimbo Orthopedic Surgery, Institutional Review Board, 5-38-41, Honcho Koganei-shi, Tokyo

Site Number	Name/Address of IRB/IEC
2004669	State Social Enterprise, HOSPITAL MENTAL DE ANTIOQUIA, [Antioquia Psychiatric Hospital], Calle 38 55-310 Bello-Colombia
2004670	NHO Okayama Medical Center IRB, Kita-ku Tamasu 1711-1, Okayama-shi, Okayama-Ken, Japan
2004671	Kawasaki Municipal Hospital Institutional Review Board, 12-1, Shinkawa-dori, Kawasaki-ku, Kawasaki-shi, Kanagawa
2004672	Laniado Hospital, 16, deuteronomy haim st., kiryat sanz, netanya, 42150
2004678	Marshfield Clinic Research Institiute Institutional Review Board, 1000N, Oak Ave, Marshfield, WI 54449-5790
2004681	Human Research Ethics Committee, Fundación Hospital Infantil Universitario de San José, Carrera 52 No. 67 A-71 PBX: 4377540
2004687	Fukuyama City Hospital Institutional Review Board, 5-23-1 Zao-cho, Fukuyama-shi, Hiroshima
2004688	KKR Sapporo Medical Center IRB, 6-3-40 Hiragishi 1-jo Toyohira-ku, Sapporo-shi, Hokkaido
2004708	EMORY UNIVERSITY Institutional Review Board, 201 Dowman Dr, Atlanta, GA 30322, United States"
2004747, 2004749	Navajo Nation Human Research Review Board, Navajo Division of Health, P. O. Box 1390, Window Rock, AZ 86515
2004748	Johns Hopkins Bloomberg School Of Public Health, Institutional Review Board Office, 615 N. Wolfe Street / Room E1100 Baltimore, Maryland 21205-2179"
2004768	Samsung Medical Center Institutional Review Board, (06351) 81 Irwon-Ro Gangnam-gu. Seoul, Korea
2004769	Yonsei University Health system, Severance Hospital, Institutional review Board, Yonsei-ro 50-1, Seodaemun-gu, Seoul, 03722
2004797	human research Protection Program of Korea University medical Center 123 Jeokgeum-ro (Gojan-dong) Danwon-gu, Ansan-si, Gyeonggi-do, 15355
2004798	Inha University Hospital Institutional Review Board, 27 Inhang-ro, Jung-gu, Incheon
2004800	Yonsei University Gangnam Severance Hospital, IRB, 2nd Floor, 235 Dogok-ro, Gangnam-gu, Seoul 06230
2005029	Fukui-ken Saiseikai Hospital Institutional Review Board, 7-1 Funabashi, Wadanaka-cho, Fukui-shi, Fukui-Ken
2005030	Institutional Review Board of Okayama City General Medical Center Okayama City Hospital, 3-20-1 Kitanagaseomotemachi, Kita-ku, Okayama-shi, Okayama
2005031	Local Independent Administrative Corporation, Hiroshima City Hospital Organization, Hiroshima City Hiroshima Citizens Hospital Institutional Review Board, 7-33 Motomachi, Naka-ku, Hiroshima-shi, Hiroshima
2005032, 2005034	Review Board of Human Rights and Ethics for Clinical Studies Institutional Review Board 13-2 Ichibancho, Chiyoda-ku, Tokyo,

Site Number	Name/Address of IRB/IEC
2005033	Aijinkai Takatsuki General Hospital IRB, 1-3-13 Kosobe-cho, Takatsuki, Osaka
2005035	Japanese Red Cross Shizuoka Hospital Institutional Review Board, 8-2 Otemachi, Aoi-ku, Shizuoka-shi, Shizuoka
2005036	JA Shizuoka Kosei Hospital Institutional Review Board, 23 Kitabancho, Aoi-ku, Shizuoka-shi, Shizuoka
2005037	Hiroshima Red Cross Hospital & Atomicbomb Survivors Hospital Institutional Review Board, 1-9-6 Sendamachi, Naka-ku, Hiroshima-shI
2005038	NHO Shikoku Medical Center for Children and Adults Institutional Review Board, 2-1-1, Senyucho, Zentsuji-shi, Kagawa, Japan
2005039	Daido Hospital Institutional Review Board, 9 Hakusuicho, Minami-ku, Nagoya, Aichi
2005049	Nagoya Ekisaikai Hospital IRB, 4-66 Shonen-Cho, Nakagawa-ku, Nagoya-Shi, Aichi
2004272, 2004044	Multicentricka eticka komise IKEM a TN,Videnska 800, Praha,140 59
2004402, 2004116	Etikprövningsmyndigheten,Box 2110,SE-750 02 Uppsala,SE-750 02
2004249, 2004298	Ethikkommission der Medizinischen Universität Graz,Auenbruggerplatz 2, Graz,8036
2004373, 2004327	Child and Adolescent Health Service (HREC), Office 5E, Perth Children's Hospital,15 Hospital Avenue Nedlands,6009
2004401, 2004399,	Ethics Committee for Multicenter Trials,8 Damyan Gruev Str., Sofia,1303
2004887, 2004896	Hospital District of Southwest Finland, Joint Municipal Authority, Ethics Committee, Turku University Hospital, T-Hospital, 6th Floor, Board meeting room A 607
2004217, 2004109, 2004216	Wits Health Consortium,31 Princess of Wales Terrace,Parktown Johannesburg, 2193
2004212, 2004106, 2004214, 2004336	Ethical Council at the MoH of RF,3 Rakhmanovsky Pereulok, Moscow,127994
2004335, 2004405, 2004048, 2004105	Northern B Health and Disability Ethics Committee,20 Aitken Street, Ministry of Health, Ethics Department, Reception - Ground Floor, Thorndon, Wellington,6011
2004034, 2004108, 2004110, 2004712	Pharma Ethics Independant Research Erthics committee,123 Amcor Road,Lyttelton Manor Pretoria,0157
2004395, 2004204, 2004277, 2004710	Lithuanian Bioethics Committee,Algirdo g. 31, Vilnius,LT-03219
2004355, 2004384, 2004682, 2004689, 2004383	NRES Committee South Central - Berkshire,South West REC Centre,Level 3, Block B Bristol,BS1 2NT
2004273, 2004045, 2004046, 2004099, 2004047, 2004274	Research Ethics Committee of the National Institute for Health Development,Hiiu 42, Tallinn,11619
2004380, 2004199, 2004331, 2004202, 2004198, 2004302	Ethics Committee for Clinical Trials of Medicinal Products,Aizkraukles street 21 - 113, Riga,LV1006
2004867, 2004868, 2004869, 2004870, 2004871, 2004872	Dr Jose Renan Esquivel Children's hospital, Panama Ave, Balboa, Calle 34 Research Bioethics Committee

Site Number	Name/Address of IRB/IEC
2004033, 2004112, 2004113, 2004114, 2004115, 2004218, 2004219, 2004311, 2004333, 2004344, 2004363, 2004369, 2004382, 2004385, 2004406, 2004675, 2004407, 2005603	Hospital Universitario Clinico San Carlos,Puerta G - Planta 4ª Norte,C/ Profesor Martin Lagos, s/n Madrid, 28040
2004674, 2004371, 2004049, 2004334, 2004206, 2004381, 2004205, 2004208, 2004350, 2004305	Komisja Bioetyczna przy Okregowej Izbie Lekarskiej w Rzeszowie,ul. Jana Dekerta 2, Rzeszów, 35-030
2004629, 2004231, 2004270, 2004299, 2004320, 2004398, 2004320	O.L.V. Ziekenhuis,Moorselbaan 164, Aalst,9300
2004303, 2004234, 2004325, 2004339, 2004343, 2004639, 2004324	Ethics Committee for Clinical Trials,8, Damyan Gruev Str.,, Sofia,1303
2004654, 2004100, 2004232, 2004374, 2004378, 2004646, 2004653, 2004676, 2005602	Comité de Protection des Personnes Ile de France VIII,Hôpital Ambroise Paré, 9 avenue Charles de Gaulle Boulogne Billancourt, 92100
2004742, 2004741, 2004313, 2004743, 2004611, 2004312, 2004644	Varsinais-Suomen sairaanhoitopiiri Eettinen toimikunta ,Kiinamyllynkatu 4-8,PL 52 Turku, 20520
