# Supplementary material for: Durability of neutralizing RSV antibodies following nirsevimab administration and elicitation of the natural immune response to RSV infection in infants
Source: Nat Med. 2023 Apr 24;29(5):1172–9. doi: 10.1038/s41591-023-02316-5 (PMC10202809; doi:10.1038/s41591-023-02316-5)
Supplement: Supplementary file 2 — Reporting Summary [file 41591_2023_2316_MOESM2_ESM.pdf]

nature portfolio

Corresponding author(s): Deidre Wilkins

Last updated by author(s): Mar 2, 2023

Reporting Summary

Nature Portfolio wishes to improve the reproducibility of the work that we publish. This form provides structure for consistency and transparency in reporting. For further information on Nature Portfolio policies, see our Editorial Policies and the Editorial Policy Checklist.

Statistics

For all statistical analyses, confirm that the following items are present in the figure legend, table legend, main text, or Methods section.

n/a	Confirmed	
☐	☒	The exact sample size (*n*) for each experimental group/condition, given as a discrete number and unit of measurement
☐	☒	A statement on whether measurements were taken from distinct samples or whether the same sample was measured repeatedly
☐	☒	The statistical test(s) used AND whether they are one- or two-sided<br>*Only common tests should be described solely by name; describe more complex techniques in the Methods section.*
☒	☐	A description of all covariates tested
☒	☐	A description of any assumptions or corrections, such as tests of normality and adjustment for multiple comparisons
☐	☒	A full description of the statistical parameters including central tendency (e.g. means) or other basic estimates (e.g. regression coefficient) AND variation (e.g. standard deviation) or associated estimates of uncertainty (e.g. confidence intervals)
☒	☐	For null hypothesis testing, the test statistic (e.g. *F*, *t*, *r*) with confidence intervals, effect sizes, degrees of freedom and *P* value noted<br>*Give P values as exact values whenever suitable.*
☐	☒	For Bayesian analysis, information on the choice of priors and Markov chain Monte Carlo settings
☒	☐	For hierarchical and complex designs, identification of the appropriate level for tests and full reporting of outcomes
☒	☐	Estimates of effect sizes (e.g. Cohen's *d*, Pearson's *r*), indicating how they were calculated

Our web collection on statistics for biologists contains articles on many of the points above.

Software and code

Policy information about availability of computer code

Data collection	No custom software or algorithms were used for data collection
Data analysis	No custom software or algorithms were used for data analysis

For manuscripts utilizing custom algorithms or software that are central to the research but not yet described in published literature, software must be made available to editors and reviewers. We strongly encourage code deposition in a community repository (e.g. GitHub). See the Nature Portfolio guidelines for submitting code & software for further information.

Data

Policy information about availability of data

All manuscripts must include a data availability statement. This statement should provide the following information, where applicable:

- Accession codes, unique identifiers, or web links for publicly available datasets
- A description of any restrictions on data availability
- For clinical datasets or third party data, please ensure that the statement adheres to our policy

Data are subject to controlled access to ensure commitment to the Responsible Data Sharing Principles as established by EFPIA and PhRMA. Any restrictions are related to ensuring the fulfillment of legal and ethical obligation to protect patients when using patient data to advance medical research. Data underlying the findings described in this manuscript may be obtained in accordance with AstraZeneca's data sharing policy described at https://astrazenecagrouptrials.pharmacm.com/ST/Submission/Disclosure.

Data for studies directly listed on Vivli can be requested through Vivli at www.vivli.org. Data for studies not listed on Vivli could be requested through Vivli at https://vivli.org/members/enquiries-about-studies-not-listed-on-the-vivli-platform/. AstraZeneca Vivli member page is also available outlining further details: https://vivli.org/ourmember/astrazeneca/.

Human research participants

Policy information about studies involving human research participants and Sex and Gender in Research.

Reporting on sex and gender

Both the Phase 2b and MELODY studies enrolled patients of either sex (as determined at birth) and no differences in outcomes were found.

Population characteristics

Baseline demographics and patient characteristics are included in Supplementary Table 1

Recruitment

Together, the phase 2b and MELODY studies enrolled infants across 4 years in both the Northern and Southern Hemispheres: the phase 2b study was performed at 164 sites in 23 countries; MELODY was performed in 160 sites in 21 countries. The MELODY population comprised healthy late preterm and term infants born ≥35 weeks 0 days gestational age who would not receive RSV prophylaxis based on the AAP or other local or national guidelines; the Phase 2b population comprised healthy late preterm and term infants born ≥29 weeks 0 days to <34 weeks 6 days gestational age.
Limitations of recruitment included: 1) infants from the phase 2b study were less diverse geographically due to restrictions related to future use consent laws for biosamples in several countries, potentially impacting the generalization of outcomes; 2) the COVID-19 pandemic created an off-cycle RSV season in 2020–2021 where lockdowns, masking, and social distancing changed the incidence and prevalence of RSV, potentially impacting efficacy estimates; 3) infants could have been exposed to RSV prior to randomization if they were aged >6 months, potentially impacting efficacy estimates.

Ethics oversight

The IRB/IEC responsible for each site reviewed and approved the final study protocols, including the final version of the informed consent form and other written information and/or materials provided to the subjects. The IRB/IEC also approved all advertising used to recruit subjects for the study. The investigator was responsible for submitting the documents to the applicable IRB/IEC, and distributing them to the study site staff.
Site Number Name/Address of IRB/IEC
Phase 2b
2002923 Pharma Ethics 123 Amcor Road Lyttelton Manor Centurion Pretoria Gauteng
2003359 MetroHealth Medical Center IRB 2500 MetroHealth Dr. Rammelkamp Bldg. Room 103 Cleveland Ohio
2002934 CEP Investiga - Instituto de Pesquisas Avenida Romeu Tortima, 739 - Cidade Universitária Campinas Sao Paulo
2002970, 2003091, 2003395, 2003007, 2003356, 2003405, 2003355, 2003354, 2003004, 2003353, 2002971, 2003124, 2003350, 2003394, 2003092, 2003348, 2003078, 2002974, 2003036, 2003346, 2003340, 2003441, 2003167, 2003338, 2003337, 2003442, 2003068, 2003038, 2003342, 2003335, 2003399, 2003086, 2003444, 2003400, 2003332, 2002976, 2003402, 2003347, 2003403, 2003125, 2003329, 2003336, 2003079, 2003401, 2003407 Copernicus Group IRB 5000 CentreGreen Way Suite 200 Cary North Carolina Adams, Gregory
2002935 CEP da Universidade Federal de Minas Gerais Avenida Presidente Antonio Carlos 6627 Unidade Administrativa II Belo Horizonte Minas Gerais Andrade
2002947 CEIC de Galicia C/ San Lázaro, s/n Secretaria Xeral. Conselleria de SanidadeDirección Santiago de Compostela La Coruña Ares
2002948 CEIC de Galicia C/ San Lázaro, s/n Secretaria Xeral. Conselleria de SanidadeDirección Santiago de Compostela La Coruña Arimany Montaña,
2003358 Medical University of South Carolina IRB 19 Hagood Avenue 6th floor, Suite 601 Charleston South Carolina
2002918 Wits Health Consortium 31 Princess of Wales Terrace Parktown Johannesburg Gauteng
2002998 Comite Etico Cientifico del Servicio de Salud Metropolitano Sur Santa Rosa 3453, Piso 1 San Miguel Santiago
2003034 CESC della Provincia di Padova Presso Azienda Ospedaliera di Padova_Via Giustiniani 1 Padova
2002939 CEP da Faculdade de Ciências Médicas e da Saúde de Juiz de Fora SUPREMA/MG Alameda Salvaterra, 200 Bairro Salvaterra Juiz de Fora Minas Gerais Bastos
2003060 CEP da Faculdade de Medicina de Botucatu - UNESP/SP Distrito de Rubião Junior Botucatu Sao Paulo
2003000 Comitato Etico per la Sperimentazione Clinica delle Provincie di Verona e Rovigo P.le Stefani, 1 Verona
2002919 Pharma Ethics 123 Amcor Road Lyttelton Manor Centurion Pretoria
2002910 Monash Health Human Research Ethics Committee (RGO) Level 2, I Block Clayton Victoria
2002953 Comité Etico Científico Servicio de Salud Valdivia Maipú 550, oficina 307 Valdivia
2002920 University of Stellenbosch Ethics Committee Faculty of Health Sciences Francie van Zijl Drive Tygerberg Cape Town Western Cape
2002956 Comité Ético Científico Servicio de Salud Metropolitano Central Victoria Subercaseaux 381, piso 4 Santiago
2003352 UTHSC IRB Office 910 Madison Suite 600 Memphis Tennessee
2003118 Memorial Health Services Research Council 2801 Atlantic Avenue Attn Research Administration Long Beach California
2002972 SUNY IRB 750 East Adams Street CWB 218G Syracuse New York
2003277 McGill University Health Center-Research Ethics Board 2155 Guy Street 2nd Floor, Room 231 Montreal Quebec
2002921 Pharma Ethics 123 Amcor Road Lyttelton Manor Centurion Pretoria Gauteng
2002973, 2003061, 2003069, 2003093, 2003331, 2003447 WIRB 1019 39th Avenue SE Suite 120 Puyallup Washington
2002905 Comité de Ética en Investigación Científica. Hospital Pediátrico Dr. Humberto Notti Bandera de Los Andes 2603 Villa Nueva Guaymallén Mendoza
2002967 R&D University Hospital Southampton NHS Foundation Trust Tremona Road, Level E, Laboratory & Pathology Block, SCBR - MP 138 Southampton Hampshire
2003320 R&D - Brighton and Sussex University Hospitals Royal Sussex County Hospital Level 5 Thomas Kemp Tower Eastern Road Brighton East Sussex
2003065 Azienda Ospedaliera Città della Salute e della Scienza di Torino Corso Bramante 88/90. Torino

2002940 Comitê de Ética em Pesquisa em Seres Humanos do Instituto de Medicina Integral Professor Fernando F Rua dos Coelhos, 300 - Boa Vista Recife Pernambuco Gomes
2002922 University of Cape Town HREC Faculty of Health Sciences Research EC E52-24 Old Main Building Groote Schuur Hospital, Observatory Cape Town Western Cape
2002941 CEP da Universidade Luterana do Brasil - ULBRA Farroupilha, 8001 - Prédio 14 - Sala 224 Bairro São José Canoas Rio Grande do Sul
2003319 R&D - Alder Hey Children's NHS Foundation Trust Eaton Road Liverpool Merseyside
2002968 R&D South West London and St George's Mental Health NHS Trust Department of Mental Health, St George's, University of London, 6th Floor, Hunter Wing, Cranmer Terrace London Greater London
2002924 Eticka komise IKEM a FTNsP Videnska 800 Praha 4 - Krc
2003343 Sharp Healthcare IRB 7930 Frost St Suite 300 San Diego California
2003341 Winthrop-University Hospital IRB 222 Station Plaza North Suite 521 Mineola New York
2002943 Comitê de Ética em Pesquisa em Seres Humanos do Hospital Pequeno Príncipe Rua Desembargador Motta, 1070 6º andar, sala do NUPE Curitiba Paraná
2003339 Marshall University Office of Research Integrity One John Marshall Drive Huntington West Virginia
2002937 Wits Health Consortium 31 Princess of Wales Terrace Parktown Johannesburg Gauteng
2002950 CEIC de Galicia C/ San Lázaro, s/n Secretaria Xeral. Conselleria de SanidadeDirección Santiago de Compostela La Coruña Martinon
2002944 CEP da Universidade de Passo Fundo/RS Universidade de Passo Fundo - BR 285, Bairro São José Passo Fundo Rio Grande do Sul
2003011 Ann & Robert H. Lurie Children's Hospital of Chicago Institutional Review Board 225 E. Chicago Avenue Box 59 Chicago Illinois
2003334, 2002975 Chesapeake IRB 7063 Columbia Gateway Drive Suite 110 Columbia Maryland
2003067 Cincinnati Children's Hospital Medical Center IRB 3333 Burnet Ave. MLC 5020 Cincinnati Ohio
2002954 Comité Ético-Científico Servicio de Salud Metropolitano Sur Oriente Av Concha y Toro 3459 Puente Alto Santiago
2003333 Childrens Hospital of Los Angeles-Committee on Clinical Investigations IRB 4650 Sunset Blvd Mail Stop #23 Dr. Andreas Reiff Los Angeles California
2003280 McGill University Health Center-Research Ethics Board 2155 Guy Street 2nd Floor, Room 231 Montreal Quebec
2003005 University of Texas at San Antonio IRB One UTSA Circle MS 4.01.82 San Antonio Texas
2002951 CEIC de Galicia C/ San Lázaro, s/n Secretaria Xeral. Conselleria de SanidadeDirección Santiago de Compostela La Coruña
2002911 Royal Children's Health Services Human Research Ethics Committee (RGO) 50 Flemington Road Parkville Victoria
2002938 Pharma Ethics 123 Amcor Road Lyttelton Manor Centurion Pretoria Gauteng
2003257 Comité Ético-Científico Servicio de Salud Viña del Mar-Quillota Calle Limache #1307 Esquina Peñablanca 2º Piso Viña del Mar Quilodran
2003035 Comitato Etico Regionale della Liguria Largo Rosanna Benzi 10 Farmacia Ospedaliera Genova
2002912 Princess Margaret Hospital for Children Ethics Committee Princess Margaret Hospital Entrance No 6, Hamilton Street Subiaco Western Australia
2003330 Arnold Palmer Medical Center Institutional Review Board 1401 Kuhl Avenue MP #21 Research Department Orlando Florida
2003328 University of Nebraska Medical Center IRB 987830 Nebaska Medical Center Omaha Nebraska
2002969 R&D - CRN Thames Valley and South Midlands 1st Floor, Manor House The John Radcliffe Hospital, Headley Way Headington Oxford Oxfordshire
2002926 Eticka komise Ustav pro peci o matku a dite Podolske nabrezi 157/36 Praha 4 - Podoli
2002909 Comité Hospitalario de Etica Necochea 675 Bahia Blanca Buenos Aires
2003274 Comité d'Ethique du CHU Ambroise Paré Boulevard Kennedy 2 Mons Van
2002955 Comité de Ética de Investigación en Seres Humanos Av. Independencia 1027, Independencia Santiago Vargas
2003009 Creighton University IRB 2500 California Plaza IRB-Biomedical Omaha Nebraska
2002966 R&D University Hospitals Bristol NHS Foundation Trust Education & Research Centre Level 3 Upper Maudlin Street Bristol Avon
2002999 Comite Etico Cientifico del Servicio de Salud Metropolitano Sur Santa Rosa 3453, Piso 1 San Miguel Santiago Villena
2002927 Eticka komise Nemocnice Havlickuv Brod Husova 2624 Havlickuv Brod Weberova,
2003448 Oklahoma University Health Sciences Center 1105 North Stonewall Avenue Oklahoma City Oklahoma
2003406 Connecticut Children's Medical Center IRB 282 Washington Street. Suite 2 K. Hartford Connecticut
2002946 University of Cape Town HREC Faculty of Health Sciences Research EC E52-24 Old Main Building Groote Schuur Hospital, Observatory Cape Town Western Cape
MELODY
2004023, 2004025, 2004027, 2004028, 2004030, 2004031, 2004032, 2004118, 2004236, 2004237, 2004239, 2004240, 2004243, 2004253, 2004255, 2004256, 2004258, 2004259, 2004260, 2004261, 2004263, 2004264, 2004267, 2004268, 2004278, 2004279, 2004280, 2004291, 2004292, 2004293, 2004314, 2004315, 2004316, 2004319, 2004323, 2004340, 2004345, 2004376, 2004386, 2004389, 2004394, 2004409, 2004613, 2004614, 2004615, 2004618, 2004624, 2004634, 2004650, 2004652, 2004656, 2004657, 2004664, 2004677, 2004679, 2004680, 2004690, 2004697, 2004699, 2004700, 2004702, 2004746, 2004873, 2005604, 2005605, 2005606 WCG IRB, 212 Carnegie Center, Suite 301, Princeton, NJ 08540, USA
2004026 The University of Oklahoma, Institutional Review Board for the Protection of Human Subjects, 1105N. Stone wall Avenue, Oklahoma City, OK73117(FWA 007961)
2004029 Nemours Office of Human Subjects Protection, Nemours/Alfred I. duPont Hospital for Children, 1600 Rockland Road, Wilmington, DE 19803
2004036 University of Cape Town Human Research Ethics Committee, DEPARTMENT OF PAEDIATRICS AND CHILD HEALTH, RED CROSS WAR MEMORIAL CHILDREN'S HOSPITAL, KLIPFONTEIN ROAD, RONDEBOSCH, 7700
2004039 Stellenbosch University Human Research Ethics Committee, Stellenbosch University, Private Bag X1, Matieland, 7602, Stellenbosch, South Africa
2004043 Servicio De Salud Metropolitano Sur Oriente Comite Etico-Cientifico, Av. Concha y Toro 3459 – Paradero 30, Vic. Mackenna
2004098 UNIVERSIDAD DE CHILE [University of Chile] – FACULTAD DE MEDICINA, HUMAN RESEARCH ETHICS COMMITTEE, Av. Libertador Bernardo O'Higgins 1058, Santiago de Chile

2004103 Comitato Etico per la Sperimentazione Clinica delle Provincie di Verona e Rovigo,P.le Stefani, 1, Verona,37126
2004111 1 Military Hospital Human Research Ethics Committee, Department of Neurology Private bag X 1026 Thaba Tswane 0143
2004117 Dept of health of Chernivtsi city council, Communal Medical Institution City Clinical Childrens' Hospital, 4 Bukovynska St, Chernivtsi, 58001
2004132 Independent Ethics Committee for Clinical Pharmacology Trials, Drug and Pharmacology Studies Foundation, LA FUNDACIÓN DE ESTUDIOS FARMACOLOGICOS Y DE MEDICAMENTOS, Pte. J. E. Uriburu 774 1º Piso Ciudad Autónoma de Buenos Aires (C1027AAP), Argentina
2004178 Landesärztekammer Baden-Württemberg, Ethik-Kommission, Liebknechtstr. 33, 70565 Stuttgart
2004182 Ethik-Kommission der Bayerischen Landesärztekammer,
Mühlbaurstr.16, D-81677 München
2004185 Ethik-Kommission an der Medizinischen Fakultaet der Universitaet Leipzig,Kaethe-Kollwitz-Strasse 82,Haus: Karl-Sudhoff-Institut Leipzig,04109
2004222, Ege University Ethics Committee,Ege Universitesi Tip Fakultesi,Klinik Arastirmalar Etik Kurulu Izmir,35100
2004227 Ministy of Health of Ukrain, Communal Non-Commercial enterprise Saint Zinaida Children's Clinical Hospital of Sumy City Council, 28 Troiiska st, Sumy, 40022
2004229 Vinnytsia regional Children's Clinical Hospital, 108 Khmelnytske shose st, Vinnytsia, 21000. Medical Ethics Commission
2004233 Universidad Pontificia Bolivariana, Calle 78 B No. 72 A 109
2004238 Institutional Review Board, Ann & Robert H. Lurie Children's Hospital of Chicago, 25 East Chicago Avenue, Chicago, Illinois
2004241 Cincinnati Children’s Hospital Institutional Review Board, 3333 Burnet Avenue | MLC 7040 | Cincinnati, OH 45229
2004281 MetroHealth Institutional Review board, 2500 MetroHealth Drive, Cleveland Ohio 44109
2004294 State Institution Academician O.M Lukyanova Institute of Pediatrics, obstetrics and gynecology of national academy of medical sciences of Ukraine, 8 P.Mayborody str Kyiv, 04050
2004295 CORPORACIÓN CIENTÍFICA PEDIÁTRICA, BIOMEDICAL RESEARCH ETHICS COMMITTEE, Calle 5 B5 No. 37 bis - 28
2004296 Ministerio de Salud,
Servicio de Salud Valdivia, Scientific Ethics Committee, V. Pérez Rosales 560 - Edificio Prales - Oficina 307 - Piso 3
2004300 Servicio de Salud Metropolitano Norte,
esearch Ethics Committee, 272,Calle Maruri 8380000 Independencia Metropolitana de Santiago
2004304 UNIVERSIDAD CES, Calle 10A No. 22 - 04 El Poblado
2004310 Creighton University office of the provost Research Compliance, 2500 California Plaza Omaha, NE 68178-0001
2004322 Communal Non-Commercial enterprise of Kharkiv Regional Council regional Children's clinical hospital, 5 Ozeryanska st Kharkiv, 61093
2004338 Odesa Regional State administration, department of health, communal enterprise, Odesa regional Children's clinical hospital, 3 Ac Vorobiov st, Odes-31, 65031
2004341 Medical University of South Carolina, 179 Ashley Ave, Charleston, SC 29425
2004351 MUHC Centre for Applied Ethics, 5100, boul. de Maisonneuve Ouest, 5th floor, Office 576, Montréal, Québec, H4A 3T2
2004359 Ethikkommission der Landesärztekammer Rheinland-Pfalz Deutschhausplatz 3 55116 Mainz
2004365 MUHC Centre for Applied Ethics, 5100, boul. de Maisonneuve Ouest, 5th floor, Office 576, Montréal, Québec, H4A 3T2
2004372 COMITÉ DE ÉTICA EN INVESTIGACIÓN VIT, Calle 24 N° 3-02 este
2004391 University of Nebraska Medical Center, 42nd and Emile Streets, Omaha, NE 68198, 402-559-4000
2004396 COMITATO ETICO DELLA FONDAZIONE POLICLINICO UNIVERSITARIO AGOSTINO GEMELLI IRCCS UNIVERSITÀ CATTOLICA DEL SACRO CUORE
2004400 Research Ethics Committee of the Health Sciences Department of the Universidad del Norte, Apartados Aéreos 1569 - 51820, Km. 5 vía Puerto Colombia
2004404 Federico Gomez Children's hospital of Mexico, National Institute of Health research office
2004616 Stony Brook University, Health Sciences Center Room 031, Stony Brook, NY 11794-8111
2004623 UBC C&W Research Ethics Board A2-141A, 950 West 28th Avenue Vancouver, BC V5Z 4H4
2004626 Soroka University Medical Center, Itzhak Rager Blv. Beer Sheva 8458900
2004632 Japanese Red cross Maebashi Hospital IRB 138-Asakuramachi, Maebashi-Shi Gunma
2004633 Ethics Commission at Communal Institution Dnipro City Children's Clinical Hospital No 5 of Dnipro City Council, 5 ivana Akinfiieva st, Dnipro 49027 Ukraine
2004648 Independent Ethics Committee for Clinical Pharmacology Trials, Drug and Pharmacology Studies Foundation, LA FUNDACIÓN DE ESTUDIOS FARMACOLOGICOS Y DE MEDICAMENTOS, Pte. J. E. Uriburu 774 1º Piso Ciudad Autónoma de Buenos Aires (C1027AAP)
Argentina
2004658 Nationwide Children's IRB, Nationwide Children’s Hospital, 700 Childrens Drive, Columbus, OH 43205
2004660 Yokosuka Kyosai Hospital IRB, 1-16 Yonegahamadori, Yokosuka Kanagawa
2004662 The University Of Tennessee, Health Science Centre Institutional Review Board, 910 Madison Avenue, Suite 600, Memphis, TN 38163
2004667, Conjoint Health Research Ethics Board, Research Services Office, 2500 University Drive, NW, Calgary AB T2N 1N4
2004668 Jimbo Orthopedic Surgery, Institutional Review Board, 5-38-41, Honcho Koganei-shi, Tokyo
2004669 State Social Enterprise, HOSPITAL MENTAL DE ANTIOQUIA, [Antioquia Psychiatric Hospital], Calle 38 55-310 Bello-Colombia
2004670 NHO Okayama Medical Center IRB, Kita-ku Tamasu 1711-1, Okayama-shi, Okayama-Ken, Japan
2004671 Kawasaki Municipal Hospital Institutional Review Board, 12-1, Shinkawa-dori, Kawasaki-ku, Kawasaki-shi, Kanagawa
2004672 Laniado Hospital, 16, deuteronomy haim st., kiryat sanz, netanya, 42150
2004678 Marshfield Clinic Research Institiute Institutional Review Board, 1000N, Oak Ave, Marshfield, WI 54449-5790
2004681 Human Research Ethics Committee, Fundación Hospital Infantil Universitario de San José, Carrera 52 No. 67 A-71 PBX: 4377540
2004687 Fukuyama City Hospital Institutional Review Board, 5-23-1 Zao-cho, Fukuyama-shi, Hiroshima
2004688 KKR Sapporo Medical Center IRB, 6-3-40 Hiragishi 1-jo Toyohira-ku, Sapporo-shi, Hokkaido
2004708 EMORY UNIVERSITY Institutional Review Board, 201 Dowman Dr, Atlanta, GA 30322, United States"

2004747, 2004749 Navajo Nation Human Research Review Board, Navajo Division of Health, P. O. Box 1390, Window Rock, AZ 86515
2004748 Johns Hopkins Bloomberg School Of Public Health, Institutional Review Board Office, 615 N. Wolfe Street / Room E1100 Baltimore, Maryland 21205-2179"
2004768 Samsung Medical Center Institutional Review Board, (06351) 81 Irwon-Ro Gangnam-gu. Seoul, Korea
2004769 Yonsei University Health system, Severance Hospital, Institutional review Board, Yonsei-ro 50-1, Seodaemun-gu, Seoul, 03722
2004797 human research Protection Program of Korea University medical Center 123 Jeokgeum-ro (Gojan-dong) Danwon-gu, Ansan-si, Gyeonggi-do, 15355
2004798 Inha University Hospital Institutional Review Board, 27 Inhang-ro, Jung-gu, Incheon
2004800 Yonsei University Gangnam Severance Hospital, IRB, 2nd Floor, 235 Dogok-ro, Gangnam-gu, Seoul 06230
2005029 Fukui-ken Saiseikai Hospital Institutional Review Board, 7-1 Funabashi, Wadanaka-cho, Fukui-shi, Fukui-Ken
2005030 Institutional Review Board of Okayama City General Medical Center Okayama City Hospital, 3-20-1 Kitanagaseomotemachi, Kita-ku, Okayama-shi, Okayama
2005031 Local Independent Administrative Corporation, Hiroshima City Hospital Organization, Hiroshima City Hiroshima Citizens Hospital Institutional Review Board, 7-33 Motomachi, Naka-ku, Hiroshima-shi, Hiroshima
2005032, 2005034 Review Board of Human Rights and Ethics for Clinical Studies Institutional Review Board 13-2 Ichibancho, Chiyoda-ku, Tokyo,
2005033 Aijinkai Takatsuki General Hospital IRB, 1-3-13 Kosobe-cho, Takatsuki, Osaka
2005035 Japanese Red Cross Shizuoka Hospital Institutional Review Board, 8-2 Otemachi, Aoi-ku, Shizuoka-shi, Shizuoka
2005036 JA Shizuoka Kosei Hospital Institutional Review Board, 23 Kitabancho, Aoi-ku, Shizuoka-shi, Shizuoka
2005037 Hiroshima Red Cross Hospital & Atomicbomb Survivors Hospital Institutional Review Board, 1-9-6 Sendamachi, Naka-ku, Hiroshima-shI
2005038 NHO Shikoku Medical Center for Children and Adults Institutional Review Board, 2-1-1, Senyucho, Zentsuji-shi, Kagawa, Japan
2005039 Daido Hospital Institutional Review Board, 9 Hakusuicho, Minami-ku, Nagoya, Aichi
2005049 Nagoya Ekisaikai Hospital IRB, 4-66 Shonen-Cho, Nakagawa-ku, Nagoya-Shi, Aichi
2004272, 2004044 Multicentricka eticka komise IKEM a TN,Videnska 800, Praha,140 59
2004402, 2004116 Etikprövningsmyndigheten,Box 2110,SE-750 02 Uppsala,SE-750 02
2004249, 2004298 Ethikkommission der Medizinischen Universität Graz,Auenbruggerplatz 2, Graz,8036
2004373, 2004327 Child and Adolescent Health Service (HREC), Office 5E, Perth Children's Hospital,15 Hospital Avenue Nedlands,6009
2004401, 2004399, Ethics Committee for Multicenter Trials,8 Damyan Gruev Str., Sofia,1303
2004887, 2004896 Hospital District of Southwest Finland, Joint Municipal Authority, Ethics Committee, Turku University Hospital, T-Hospital, 6th Floor, Board meeting room A 607
2004217, 2004109, 2004216 Wits Health Consortium,31 Princess of Wales Terrace,Parktown Johannesburg, 2193
2004212, 2004106, 2004214, 2004336 Ethical Council at the MoH of RF,3 Rakhmanovsky Pereulok, Moscow,127994
2004335, 2004405, 2004048, 2004105 Northern B Health and Disability Ethics Committee,20 Aitken Street, Ministry of Health, Ethics Department, Reception - Ground Floor, Thorndon, Wellington,6011
2004034, 2004108, 2004110, 2004712 Pharma Ethics Independant Research Erthics committee,123 Amcor Road,Lyttelton Manor Pretoria,0157
2004395, 2004204, 2004277, 2004710 Lithuanian Bioethics Committee,Algirdo g. 31, Vilnius,LT-03219
2004355, 2004384, 2004682, 2004689, 2004383 NRES Committee South Central - Berkshire,South West REC Centre,Level 3, Block B Bristol,BS1 2NT
2004273, 2004045, 2004046, 2004099, 2004047, 2004274 Research Ethics Committee of the National Institute for Health Development,Hiiu 42, Tallinn,11619
2004380, 2004199, 2004331, 2004202, 2004198, 2004302 Ethics Committee for Clinical Trials of Medicinal Products,Aizkraukles street 21 - 113, Riga,LV1006
2004867, 2004868, 2004869, 2004870, 2004871, 2004872 Dr Jose Renan Esquivel Children's hospital, Panama Ave, Balboa, Calle 34 Research Bioethics Committee
2004033, 2004112, 2004113, 2004114, 2004115, 2004218, 2004219, 2004311, 2004333, 2004344, 2004363, 2004369, 2004382, 2004385, 2004406, 2004675, 2004407, 2005603 Hospital Universitario Clinico San Carlos,Puerta G - Planta 4ª Norte,C/ Profesor Martin Lagos, s/n Madrid, 28040
2004674, 2004371, 2004049, 2004334, 2004206, 2004381, 2004205, 2004208, 2004350, 2004305 Komisja Bioetyczna przy Okregowej Izbie Lekarskiej w Rzeszowie,ul. Jana Dekerta 2, Rzeszów, 35-030
2004629, 2004231, 2004270, 2004299, 2004320, 2004398, 2004320 O.L.V. Ziekenhuis,Moorselbaan 164, Aalst,9300
2004303, 2004234,
2004325, 2004339,
2004343, 2004639,
2004324 Ethics Committee for Clinical Trials,8, Damyan Gruev Str.,, Sofia,1303
2004654, 2004100, 2004232, 2004374, 2004378, 2004646, 2004653, 2004676, 2005602 Comité de Protection des Personnes Ile de France VIII,Hôpital Ambroise Paré, 9 avenue Charles de Gaulle Boulogne Billancourt, 92100
2004742, 2004741, 2004313, 2004743, 2004611, 2004312, 2004644 Varsinais-Suomen sairaanhoitopiiri Eettinen toimikunta ,Kiinamyllynkatu 4-8,PL 52 Turku, 20520

Note that full information on the approval of the study protocol must also be provided in the manuscript.

Field-specific reporting

Please select the one below that is the best fit for your research. If you are not sure, read the appropriate sections before making your selection.

☒ Life sciences ☐ Behavioural & social sciences ☐ Ecological, evolutionary & environmental sciences

For a reference copy of the document with all sections, see nature.com/documents/nr-reporting-summary-flat.pdf

Life sciences study design

All studies must disclose on these points even when the disclosure is negative.

Sample size	The sample size of 1,500 is necessary based on advice from the US FDA requesting that 1,000 preterm infants be exposed to nirsevimab in this Phase 2b study. This sample size has approximately > 99% power to detect 70% relative risk reduction, assuming a placebo group medically attended RSV LRTI incidence of 8%. Power calculations are based on Poisson regression model with robust variance (Zou Am J Epidemiol 2004;159:702-706) comparing nirsevimab 50 mg versus placebo, with 2-sided, $\alpha = 0.049$ (due to 0.001 alpha spend at the interim analysis; refer to Interim Analysis Section 4). The 70% relative risk reduction assumption is based on a placebo-controlled study in Native American infants in which there was 87% relative reduction in the incidence of RSV hospitalization (11.3% placebo; 1.5% motavizumab; $p < 0.001$) and 71% relative reduction in the incidence of outpatient RSV LRTI (10.0% placebo; 2.9% motavizumab; $p < 0.001$) in infants who received motavizumab prophylaxis (O'Brien et al. Lancet Infect Dis 2015;15:1398-1408) In order to evaluate risk, a sample size of 1,000 subjects exposed to nirsevimab will provide a 90% probability of observing at least one AE if the true event rate is 0.2%; if no AEs are observed, this study provides 95% confidence that the true event rate is < 0.3%.<br>With 3000 subjects, MELODY had at least 99% power for the primary efficacy endpoint. Analysis of the primary efficacy endpoint based on the 1490 subjects randomised prior to the pause of the enrolment due to the COVID-19 pandemic, still allowed the study to be sufficiently powered. More specifically, the sample size of approximately 1500 subjects in the Primary Cohort has at least 99% power to detect a 70% RRR, assuming an 8% incidence of MA RSV LRTI in the placebo group. Power calculations were based on a Poisson regression model with robust variance (Zou Am J Epidemiol 2004;159:702-706) comparing nirsevimab versus placebo, with 2-sided, $\alpha = 0.05$. The assumption of 8% incidence is supported both by literature (Paramore et al. Pediatr Pulmonol 2010;45:578-584) and the observed placebo incidence rate (9.6%) in Study 3. The 70% RRR assumption is based on Study 3 in which there was a 70% RRR in the incidence of MA RSV LRTI (9.5% placebo, 2.6% nirsevimab; $p < 0.001$) and 79% RRR in the incidence of MA RSV LRTI with hospitalisation (4.1% placebo, 0.8% nirsevimab; $p < 0.001$) in subjects who received nirsevimab prophylaxis. In addition, the assumption is supported by a placebo-controlled study in Native American term infants in which there was a 71% relative reduction in the incidence of outpatient RSV LRTI (10.0% placebo, 2.9% motavizumab; $p < 0.001$) and 87% relative reduction in the incidence of RSV hospitalisation (11.3% placebo, 1.5% motavizumab; $p < 0.001$) in infants who received motavizumab prophylaxis (O'Brien et al. Lancet Infect Dis 2015;15:1398-1408). In the event that the incidence rate in the placebo group decreased due to the impact of the COVID-19 pandemic (eg, social distancing), the sample size of 1500 provided at least 90% power to detect a 70% RRR if the placebo incidence rate is 4% or higher.
Data exclusions	No data were excluded from the analysis
Replication	The RSV neutralizing antibody and 5-plex ECL based serology assay were validated by measurements of range, precision, dilutional linearity, selectivity, relative accuracy and ruggedness. It is the view of the authors that these measurements verify the reproducibility of the experimental findings. Given the inclusion of ruggedness in the validation, experimental replication was not performed.
Randomization	An interactive web response system was used for randomization to a treatment group and assignment of blinded investigational product kit numbers in both studies. Pooling was based on the treatment groups generated at randomization as no further treatment was administered.
Blinding	The subject/legal representative, investigators and site staff were blinded with regard to the treatment received.

Reporting for specific materials, systems and methods

We require information from authors about some types of materials, experimental systems and methods used in many studies. Here, indicate whether each material, system or method listed is relevant to your study. If you are not sure if a list item applies to your research, read the appropriate section before selecting a response.

Materials & experimental systems

n/a	Involved in the study	
☐	☒	Antibodies
☐	☒	Eukaryotic cell lines
☒	☐	Palaeontology and archaeology
☒	☐	Animals and other organisms
☐	☒	Clinical data
☒	☐	Dual use research of concern

Methods

n/a	Involved in the study	
☒	☐	ChIP-seq
☒	☐	Flow cytometry
☒	☐	MRI-based neuroimaging

Antibodies

Antibodies used	SULFO-TAG-labelled monoclonal anti-human IgG detection antibody, clone 2A11
Validation	The detection antibody characterization, conjugation and performance optimization was performed by Meso Scale Diagnostics. The detection antibody was used in the assay at a concentration of 1 µg/mL.

Eukaryotic cell lines

Policy information about cell lines and Sex and Gender in Research

Cell line source(s)	Vero cells - expanded from client provided vial lot # VEROp.123-070827, to create Vero Working Cell Bank lot # VERO p128-190710, frozen on 24Jul2019 expiry 24Jul2039
Authentication	2 cryovials of the working cell bank were sent to a 3rd party vendor (IDEXX) to be tested via PCR and culturing to confirm species identify. Report showed PCR and genetic evaluation were confirmed to be of African Green Monkey origin with no interspecies contamination
Mycoplasma contamination	A cryovial post-banking of the Vero Working Cell Bank was sent to a 3rd party vendor (IDEXX) to be tested for mycoplasma and other contaminates. Report showed that cell line was negative for Mycoplasma sp., bacterial growth, and fungal growth.
Commonly misidentified lines (See ICLAC register)	N/A

Clinical data

Policy information about clinical studies

All manuscripts should comply with the ICMJE guidelines for publication of clinical research and a completed CONSORT checklist must be included with all submissions.

Clinical trial registration	Phase 2b: NCT02878330; MELODY: NCT03979313
Study protocol	Phase 2b: https://clinicaltrials.gov/ct2/show/NCT02878330. MELODY: the protocol is not yet publicly available as the study is ongoing
Data collection	Phase 2b was conducted at 164 sites in 23 countries across the globe between November 3, 2016 (study start date) and December 6, 2018 (actual study completion date).<br>The MELODY primary cohort was conducted at 160 sites in 21 countries across the globe between July 23, 2019 (study start date) and March 11, 2020 (enrolment pause due to COVID-19 pandemic) with a final estimated completion date of March 21, 2023.

Outcomes

Phase 2b:
Primary outcome measures:
Number of participants with medically attended respiratory syncytial virus (RSV) confirmed lower respiratory tract infection (LRTI) [Time frame: from Day 1 through Day 151]
The determination of medically attended RSV LRTI is based on objective clinical LRTI criteria and RSV test results obtained from analysing the respiratory secretions using a validated RSV real time reverse transcriptase-polymerase chain reaction (RT-PCR) assay for the detection of RSV A or RSV B subtypes. Criteria for LRTI included documented physical exam findings of rhonchi, rales, crackles, or wheeze and any of the following: increased respiratory rate at rest (for age 2 months: ≥60 breaths/min; 2-6 months: ≥50 breaths/min; and for >6 months - 2 years, ≥40 breaths/min), or hypoxemia (in room air - oxygen saturation <95% at altitudes ≤1800 meters or <92% at altitudes >1800 meters), or clinical signs of severe respiratory disease or dehydration secondary to inadequate oral intake due to respiratory distress (need for intravenous fluid).
Secondary outcome measures:
1. Number of participants hospitalized due to RSV confirmed LRTI [Time frame: from Day 1 through Day 151]
An RSV hospitalization is defined as either 1) a respiratory hospitalization with a positive RSV test within 2 days of hospitalization (primary) or 2) new onset of respiratory symptoms in an already hospitalized child, with an objective measure of worsening respiratory status and positive RSV test (nosocomial).
2. Number of participants with treatment emergent adverse events (TEAEs) and treatment emergent serious adverse events (TESAEs) [Time frame: from Day 1 through Day 361]
3. Number of participants with adverse events of special interest (AESIs) and new onset chronic diseases (NOCDs) [Time Frame: From Day 1 through Day 361]
4. Serum concentration of nirsevimab [time frame: Days 91, 151, and 361]
5. Elimination half-life (t1/2) of nirsevimab [Time frame: Day 91 through Day 361]
6. Number of participants with positive anti-drug antibodies (ADA) to nirsevimab [Time frame: Days 91, 151, and 361]

MELODY:
Primary outcome measures:
1. Incidence of medically attended LRTI due to RT-PCR confirmed RSV [Time frame: 150 days post-dose]
The incidence of RSV LRTI (inpatient and outpatient) 150 days post dose will be based on RSV test results (performed centrally via RT-PCR) and objective clinical LRTI criteria and will be presented by treatment group. The relative risk reduction of nirsevimab over placebo in preventing RSV LRTI will be estimated from model.
Secondary outcome measures:
1. Incidence of hospitalization due to RT-PCR confirmed RSV [Time frame: 150 days post-dose]
The incidence of RSV hospitalization 150 days post dose will be presented by treatment group. The relative risk reduction of nirsevimab over placebo in preventing RSV hospitalization will be estimated from model.
2. Safety and tolerability of nirsevimab as assessed by the occurrence of all TEAEs and TESAE [Time frame: 360 days post-dose]
Other safety assessments will include the occurrence of AESIs and NOCDs.
3. Single-dose serum concentrations of nirsevimab [Time frame: 360 days post-dose]
Nirsevimab serum concentration levels will be assessed by mean serum concentration of nirsevimab at pre-specified timepoints and tabulated by treatment group.
4. Incidence of ADA to nirsevimab in serum [Time frame: 360 days post-dose]
The incidence of ADA to nirsevimab will be assessed and summarized by percentage of subjects that are ADA positive by treatment

group.
